# GATA-Dependent Glutaminolysis Drives Appressorium Formation in *Magnaporthe oryzae* by Suppressing TOR Inhibition of cAMP/PKA Signaling

**DOI:** 10.1371/journal.ppat.1004851

**Published:** 2015-04-22

**Authors:** Margarita Marroquin-Guzman, Richard A. Wilson

**Affiliations:** Department of Plant Pathology, University of Nebraska-Lincoln, Lincoln, Nebraska, United States of America; Purdue University, UNITED STATES

## Abstract

Fungal plant pathogens are persistent and global food security threats. To invade their hosts they often form highly specialized infection structures, known as appressoria. The cAMP/ PKA- and MAP kinase-signaling cascades have been functionally delineated as positive-acting pathways required for appressorium development. Negative-acting regulatory pathways that block appressorial development are not known. Here, we present the first detailed evidence that the conserved Target of Rapamycin (TOR) signaling pathway is a powerful inhibitor of appressorium formation by the rice blast fungus *Magnaporthe oryzae*. We determined TOR signaling was activated in an *M*. *oryzae* mutant strain lacking a functional copy of the GATA transcription factor-encoding gene *ASD4*. Δ*asd4* mutant strains could not form appressoria and expressed *GLN1*, a glutamine synthetase-encoding orthologue silenced in wild type. Inappropriate expression of *GLN1* increased the intracellular steady-state levels of glutamine in Δ*asd4* mutant strains during axenic growth when compared to wild type. Deleting *GLN1* lowered glutamine levels and promoted appressorium formation by Δ*asd4* strains. Furthermore, glutamine is an agonist of TOR. Treating Δ*asd4* mutant strains with the specific TOR kinase inhibitor rapamycin restored appressorium development. Rapamycin was also shown to induce appressorium formation by wild type and Δ*cpka* mutant strains on non-inductive hydrophilic surfaces but had no effect on the MAP kinase mutant Δ*pmk1*. When taken together, we implicate Asd4 in regulating intracellular glutamine levels in order to modulate TOR inhibition of appressorium formation downstream of cPKA. This study thus provides novel insight into the metabolic mechanisms that underpin the highly regulated process of appressorium development.

## Author Summary

Many fungal pathogens destroy important crops by first gaining entrance to the host using specialized appressorial cells. Understanding the molecular mechanisms that control appressorium formation could provide new routes for managing severe plant diseases. Here, we describe a previously unknown regulatory pathway that suppresses appressorium formation by the rice pathogen *Magnaporthe oryzae*. We provide evidence that a mutant *M*. *oryzae* strain, unable to form appressoria, accumulates intracellular glutamine that, in turn, inappropriately activates a conserved signaling pathway called TOR. Reducing intracellular glutamine levels, or inactivating TOR, restored appressorium formation to the mutant strain. TOR activation is thus a powerful inhibitor of appressorium formation and could be leveraged to develop sustainable mitigation practices against recalcitrant fungal pathogens.

## Introduction

Fungal pathogens cause some of the most devastating crop diseases and constitute globe-wide challenges to socioeconomic growth and food security. To facilitate entry into their hosts, many filamentous pathogens form highly specialized infection structures, known as appressoria, on the leaf surface [1 – 3]. Appressoria breach the host cuticle and allow access to the underlying epidermal cells. Appressoria have varying morphologies that range from undifferentiated germ tube swellings to discrete dome-shaped cells separated from the germ tube tip by septa [1, 4, 5]. In addition to facilitating plant invasion, appressoria can act as sites of effector delivery and thus mediate the molecular host-pathogen interaction [6, 7]. Despite their widespread occurrence and long-acknowledged importance to plant health, detailed mechanistic descriptions of the regulatory pathways necessary for appressorium formation are limited to two molecular pathways, the cAMP/ PKA- and MAP kinase—signaling cascades [2, 5, 8 – 10].

One filamentous pathogen that has been widely studied as a model to understand the molecular biology of appressorium development is the rice blast fungus *Magnaporthe oryzae* [5, 10]. This pathogen is notable for the serious threat it poses to rice production worldwide, destroying 10–30% of the global rice harvest each year. Infection begins when a three-celled spore of *M*. *oryzae* adheres to the surface of a rice leaf and germinates. At 4 hours post inoculation (hpi), the germ tube hooks and begins to swell. By 8 hpi the swelling has developed into a dome-shaped appressorium that becomes melanized, pressurized, and infection competent by 16–24 hpi [3, 5, 11]. The tightly regulated morphological transitions that occur during appressorium development are dependent on a range of external cues, including surface hardness and hydrophobicity [12, 13], that act to trigger internal regulatory processes such as adenylate cyclase activation and cAMP production [5, 8, 9]. cAMP acts by binding the regulatory subunit of protein kinase A (PKA) to release the protein kinase A catalytic subunit (cPKA). Genetic lesions in the cAMP/ PKA signaling pathway significantly reduce appressorium formation and those that do form are small and non-functional [5, 14, 15]. Appressorium formation can be remediated by the addition of cAMP when pathway mutations occur upstream of PKA. Moreover, activating cPKA by exogenous cAMP can induce appressorium formation in wild type strains (WT) on non-inductive hydrophilic surfaces [14]. Another internal regulatory process that has been well documented to control appressorium morphogenesis is the Pmk1 MAP kinase signaling cascade. The MAP kinase orthologue of Fus3/Kss1, Pmk1, is essential for appressorial formation and works in a MAP kinase cascade instigated by hydrophobicity and cutin monomer sensing [9, 10, 16]. Disruptions to MAP kinase signaling abolish the initiation of appressorium formation, and the germ tubes of Δ*pmk1* mutants remain undifferentiated [17]. Thus, the positive-acting cAMP/ PKA and MAP kinase morphogenetic regulatory cascades are integral to appressorium initiation and development.

Here, we present genetic and biochemical evidence for a previously unknown, negative-acting regulator that inhibits appressorium formation downstream of cPKA. In a previous study [18], we showed that the GATA family [19] transcription factor Asd4 was essential for sporulation, optimal growth on undefined complete media (CM) and appressorium formation [18]. Spores of Δ*asd4* mutant strains lacking a functional *ASD4* allele due to homologous gene replacement produced germ tubes that could not elaborate appressoria at the apical tips. However the mechanisms involved, and any relationship of the GATA factor Asd4 to cAMP/ PKA and MAP kinase signaling, were unknown [18]. Here, we show that Asd4 regulates the expression of genes involved in nitrogen assimilation and glutaminolysis in order to modulate intracellular glutamine pools. Elevated intracellular pools of glutamine in Δ*asd4* mutant strains activated the target of rapamycin (TOR) nutrient-sufficiency signaling pathway [20] and prevented appressorium formation. Remediating glutamine levels in Δ*asd4* mutant strains by genetic manipulation, or bypassing the elevated glutamine signal using the specific TOR inhibitor rapamycin, promoted appressorium formation by Δ*asd4* mutant strains. Rapamycin treatment also induced appressorium formation in Δ*cpkA* mutant strains. However, cAMP treatment did not restore appressorium formation to Δ*asd4* mutant strains, and rapamycin treatment did not stimulate appressorium formation in Δ*pmk1* mutant strains. When considered together, the results presented here implicate Asd4, glutamine metabolism and TOR as fundamental but previously unknown regulators of plant disease that act on the cAMP/ cPKA signaling pathway to control appressorium formation.

## Results

### Asd4 is involved in nitrogen metabolism

**Fig 1:**
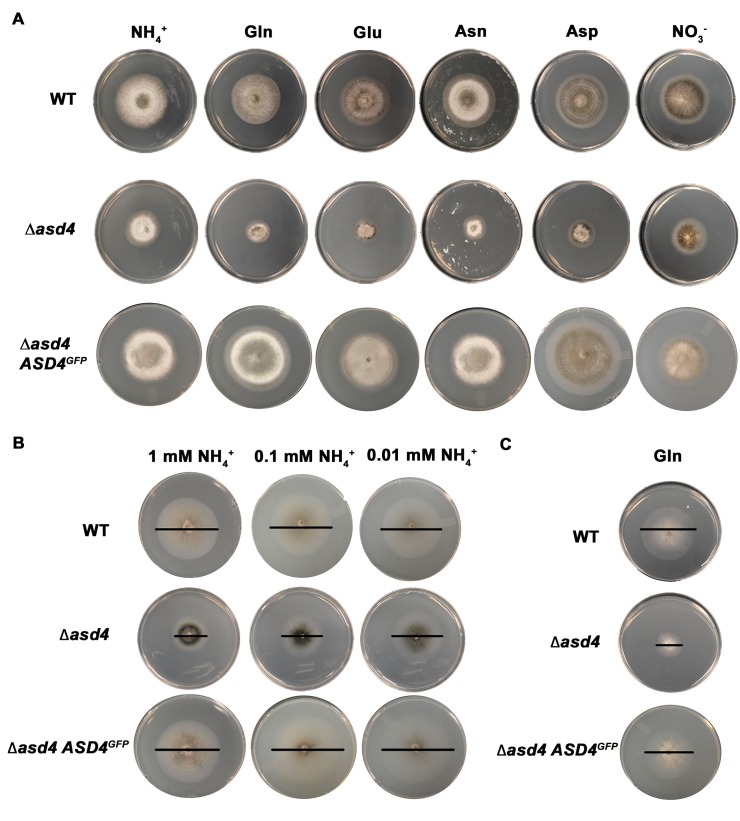
*ASD4* is involved in nitrogen assimilation. (A) Δ*asd4* mutant strains were grown for 10 days on minimal media (MM) containing 1% (w/v) glucose (GMM) and 10 mM of the indicated sole nitrogen sources. L-isomers were used throughout this study. (B) After 10 days, Δ*asd4* growth was not further attenuated on GMM containing low concentrations of ammonium as the sole nitrogen. (C) Strains were grown for 10 days on MM containing 10 mM L-glutamine as the sole carbon and nitrogen source. WT = wild type Guy11 isolate, Δ*asd4 ASD4GFP* = Δ*asd4* complementation strain expressing Asd4 fused to GFP. Bars are added as a visual aid to the plate images in (B) and (C) to demarcate colony size.

To investigate the role of Asd4 in appressorium formation, we first turned our attention to the connection between Asd4 function and optimal axenic growth on plate media. In common with other fungi, *M*. *oryzae* preferentially utilizes glucose and ammonium (NH_4_^+^) over other carbon and nitrogen sources [21, 22]. When grown on defined minimal media containing 1% (w/v) glucose (GMM) and 10 mM NH_4_^+^ as the sole carbon and nitrogen source, respectively, Δ*asd4* mutant strains, compared to the Guy11 wild type (WT) isolate used in our studies, were reduced for growth **(Fig 1A and S1 Table).** Reduced growth on GMM with 10 mM NH_4_^+^ was similarly observed for Δ*asd4* mutant strains generated from the *M*. *oryzae* reference isolate 70–15 (**S1A Fig**) and, consistent with previous observations, loss of *ASD4* function in 70–15 also abolished appressorium formation (**S1B Fig**). Reduced growth on NH_4_^+^- media was not observed, however, for a Δ*asd4 ASD4* ^*GFP*^ complementation strain expressing Asd4 fused to GFP from its native promoter in the Guy11-derived Δ*asd4* mutant background (**Fig 1A**). The Δ*asd4 Asd4* ^*GFP*^ complementation strain was also restored for appressoria formation on hydrophobic surfaces (**S1C Fig**) and Asd4^GFP^ localized, as expected for a transcription factor, to the appressorial nucleus (**S1D Fig**). Thus, the role of Asd4 in growth and appressorium formation is not idiosyncratic to the Guy11 isolate (nor does the Δ*asd4* phenotype result from off-target gene deletion effects) but is rather a fundamental function of this GATA factor in *M*. *oryzae*.

Additional plate testing revealed that Δ*asd4* mutant strains (but not WT or Δ*asd4 ASD4* ^*GFP*^ complementation strains) were also defective for growth on glucose MM (GMM) regardless of the nitrogen source, including compounds less preferred than NH_4_^+^ such as amino acids and nitrate (NO_3_^-^) (**Fig 1A** and **S1 Table**). Δ*asd4* radial growth was not further restricted by low (< 10 mM) concentrations of NH_4_^+^, amino acids or GABA as sole nitrogen sources (**Fig 1B and S1 Table**), suggesting Asd4 is not involved in nitrogen uptake. Asd4 is also not involved in sugar utilization because we observed poor growth of Δ*asd4* mutant strains on MM with L-glutamine as a sole carbon and nitrogen source (**Fig 1C**); on MM lacking glucose but containing the less preferred, derepressing sugar xylose with L-glutamine or L-glutamate as a nitrogen source (**S2 Table**); and on MM with L-glutamine or L-GABA as sole carbon sources and with NH_4_^+^ as a nitrogen source (**S2 Table**). Taken together, these observations indicate Asd4 is not required for nitrogen uptake (**Fig 1B**) or sugar metabolism (**S2 Table**), but is required for nitrogen metabolism (**Fig 1A**) including glutaminolysis (**Fig 1C**).

### Asd4 regulates the expression of genes involved in nitrogen assimilation and glutaminolysis

**Fig 2:**
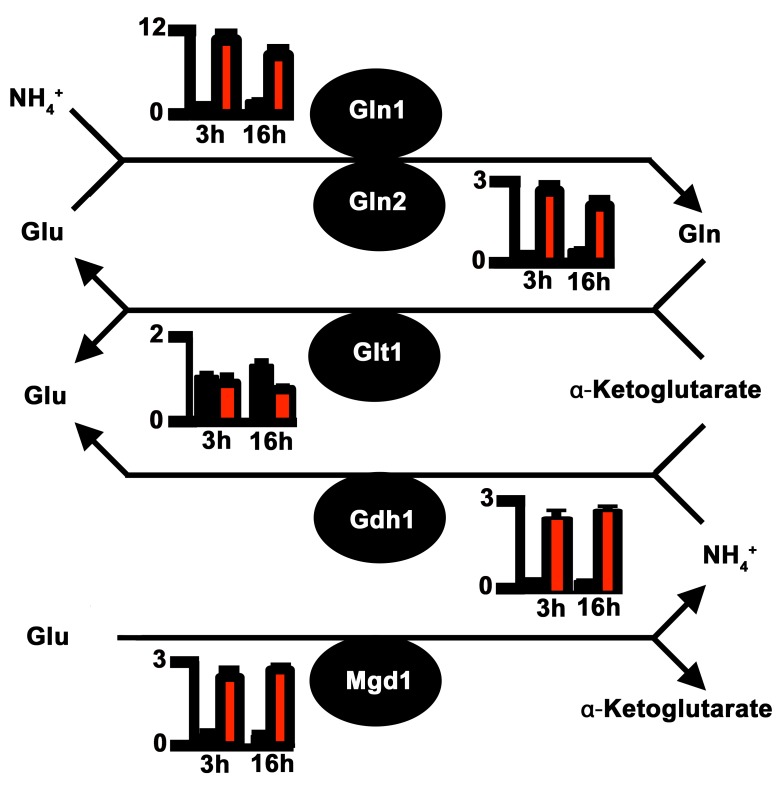
Proposed pathway of nitrogen assimilation and glutaminolysis in *M*. *oryzae* based on sequence homology. Nitrogen is assimilated and distributed via glutamine synthetase (Gln1 and Gln2) and glutamate synthase (Glt1) or anabolic NADP-dependent glutamate dehydrogenase (Gdh1) and Gln1-2 [24, 25]. Glutaminolysis [26] involves NAD-dependent glutamate dehydrogenase (Mgd1). Enzymes are depicted as solid circles with the adjacent bar graphs showing the relative expression levels of the encoding genes in WT (black bar) and Δ*asd4* mutant strains (red bar) following 3 h and 16 h growth in GMM with 10 mM NH_4_^+^ as the sole nitrogen source. Results were normalized against the expression of the β-tubulin gene *TUB2*. Values are the average of three results from at least two independent biological replicates. Error bars are standard deviation.

Impaired nitrogen assimilation in Δ*asd4* mutant strains could account for its poor growth on all the nitrogen sources tested regardless of the carbon source (**Fig 1A and 1C**). Based on sequence homology, we identified genes in the *M*. *oryzae* genome [23] encoding likely components of the nitrogen assimilatory and glutaminolytic pathways [24 – 26], including two glutamine synthetase-encoding orthologues, *GLN1* and *GLN2* (**Fig 2 and S3 Table**). To determine the expression profiles of these genes we used quantitative real-time PCR (qPCR) to analyze RNA extracted from WT and Δ*asd4* mutant strains grown in liquid shake GMM with 10 mM NH_4_^+^ for 3 and 16 h [21]. Loss of Asd4 function induced the expression of *GLN1* and up-regulated the expression of *GLN2*, *GDH1* and *MGD1* compared to WT (**Figs 2 and S2**). Thus, genes for assimilating and metabolizing nitrogen are misregulated in Δ*asd4* mutant strains when compared to WT.

To understand how the gene expression perturbations in **Fig 2**might affect nitrogen metabolism, we measured the steady-state concentrations of amino acids in WT and Δ*asd4* mycelia following growth in GMM with 10 mM NH_4_^+^ for 16 h using LC-MS/MS (**Table 1**). Steady-state intracellular pools of glutamine were significantly (*Student’s t-test* p ≤ 0.05) increased in Δ*asd4* mycelial extracts compared to WT (**Table 1**). This suggests that defects in glutaminolysis (**Fig 1C**) and/ or the misregulation of nitrogen assimilation genes (**Fig 2)** significantly affected glutamine biosynthesis and turnover in Δ*asd4* mutant strains. The concentrations of other intracellular amino acids pools were also altered in Δ*asd4* mutant strains under these growth conditions. For instance, **Table 1**shows that steady-state intracellular pools of aspartate, alanine and arginine were reduced, while asparagine and valine levels were increased, in Δ*asd4* mycelia compared to WT. Collectively, these results demonstrate that glutamine turnover and the distribution of assimilated nitrogen into other nitrogenous compounds is perturbed in Δ*asd4* mutant strains compared to WT. These observations are consistent with our plate tests (**Fig 1A, 1B and 1C)** showing Δ*asd4* strains were impaired in nitrogen source utilization and glutaminolysis.

**Table 1:** aTRAQ values for mycelial amino acid concentrations following growth on minimal media with 1% (w/v) glucose and 10 mM NH_4_^+^.

Strains			WT		*Δasd4*		*Δasd4 Δgln1*	
Amino acids	p-value	LSD^a^	Mean (μmole/g)^b^	SD^c^	Mean (μmole/g)^b^	SD^c^	Mean (μmole/g)^b^	SD^c^
Alanine	0.001	12.455	75.53	10.11	47.89	2.86	41.69	2.48
Arginine	0.003	7.113	24.44	5.59	12.31	2.29	7.95	1.22
Asparagine	0.009	1.897	5.15	0.94	8.67	0.95	6.00	0.97
Aspartate	0.031	0.577	1.23	0.38	0.47	0.17	0.53	0.28
Glutamate	0.404	-	6.04	2.70	8.98	0.62	6.13	1.58
Glutamine	0.002	19.398	40.41	10.00	91.6	6.00	56.95	7.72
Glycine	0.030	0.438	2.25	0.24	1.88	0.18	1.60	0.23
Histidine	0.001	0.425	2.51	0.28	1.71	0.19	1.26	0.14
Isoleucine	0.009	0.628	1.52	0.18	2.67	0.17	2.48	0.48
Leucine	0.549	-	1.47	0.17	1.63	0.29	1.74	0.35
Lysine	0.018	0.643	2.40	0.37	2.46	0.08	1.49	0.40
Methionine	0.710	-	0.07	0.02	0.05	0.04	0.07	0.03
Phenylalanine	0.574	-	0.50	0.07	0.52	0.04	0.56	0.09
Proline	0.149	-	2.76	0.47	3.93	0.46	4.07	1.14
Serine	0.114	1.988	4.78	0.54	4.12	0.42	3.81	0.44
Threonine	0.0002	0.587	3.37	0.50	5.65	0.03	4.09	0.06
Tryptophan	0.234	-	0.26	0.04	0.29	0.00	0.31	0.04
Tyrosine	0.848	-	3.98	0.60	4.71	1.11	5.09	3.91
Valine	0.004	0.854	3.89	0.43	5.89	0.48	4.63	0.37

^a^LSD = Fisher's Least Significant Difference.

^b^Values correspond to the average of three independent repetitions.

^c^Standard deviation.

### Asd4-dependent silencing of the glutamine synthetase—Encoding gene *GLN1* is essential for appressorium formation

**Fig 3:**
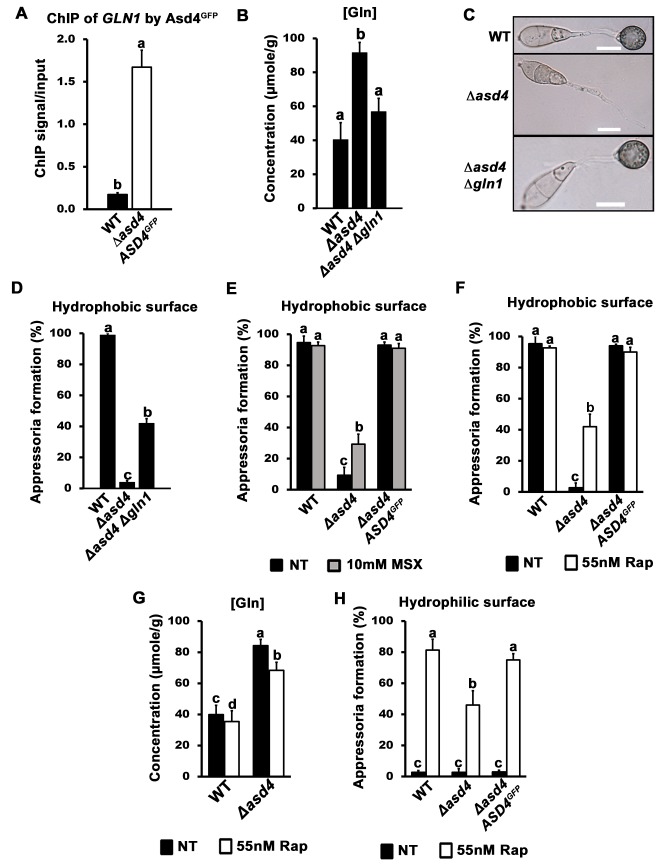
A real or perceived reduction in intracellular glutamine levels restores appressorium formation to Δ*asd4* mutant strains. (A) Asd4 physically interacts with the *GLN1* promoter *in vivo* following growth on GMM with 10 mM NH_4_^+^ as the sole nitrogen source. ChIP was performed using Anti-GFP. The eluted *GLN1* DNA signal, normalized against the input control, was significantly (*Student’s t-test* p ≤ 0.05) enriched in Δ*asd4 ASD4GFP* ChIP samples compared to WT controls lacking Asd4^GFP^. (B) Steady-state intracellular concentrations of glutamine were quantified using LC-MS/MS analysis of amino acid extracts from dried fungal mycelia of the indicated strains following growth on GMM + 10 mM NH_4_^+^ media. Values are the average of three independent biological replicates. (C) Appressorial development of WT, *Δasd4* and *Δasd4 Δgln1* strains on artificial hydrophobic surfaces (coverslips) at 24 hpi. Scale bar is 10 μm. (D) Appressorial formation rates of WT, *Δasd4* and *Δasd4 Δgln1* mutant strains on artificial hydrophobic surfaces (coverslips). (E) Treatment of Δ*asd4* spores with the glutamine synthetase inhibitor 10 mM L-methionine sulphoximine (MSX) partially remediated appressorium formation. (F) *Δasd4* mutant strains formed appressoria at 24 hpi on artificial hydrophobic surfaces following treatment with 55 nM rapamycin (Rap). (G) Steady-state intracellular concentrations of glutamine were quantified following growth of the indicated strains on GMM + 10 mM NH_4_^+^ media with and without 55 nM rapamycin. Values are the average of three independent biological replicates. (H) Treating spore suspensions with 55 nM rapamycin induced appressorium formation by WT and Δ*asd4* mutant strains on non-inductive hydrophilic surfaces. NT is untreated. (A-B, D-H) Error bars are standard deviation. Bars with different letters are significantly different (*Student’s t-test* p ≤ 0.05). (D-F, H) Values are the average of the number of appressoria formed at 24 hpi from 50 spores per coverslip, repeated in triplicate.

We hypothesized that the misregulation of *GLN1/2*, *MGD1* and *GDH1* gene expression might account for the accumulation of glutamine in Δ*asd4* mutant strains compared to WT. Of particular note, *GLN1* expression was detected in Δ*asd4* mutant strains on NH_4_^+^ media but not in WT (**Figs 2 and S2**). To determine how *GLN1* expression might contribute to the observed Δ*asd4* phenotypes, we first characterized how *GLN1* was expressed under different developmental and growth conditions. We found that, in contrast to *GLN2*, *GLN1* gene expression was not detected in WT during appressoria development [27] (**S3A Fig**). Furthermore, our qPCR transcript analysis showed that, unlike *GLN2*, *GLN1* was not expressed during early *in planta* colonization by WT (**S3B Fig**), or during the growth of WT on a range of nitrogen sources in addition to NH_4_^+^ (**S3C Fig**). However, *GLN1* was highly expressed in Δ*asd4* mutant strains compared to WT on all the nitrogen sources tested (**S3C Fig**). These expression data prompted us to perform chromatin immunoprecipitation (ChIP) in order to determine whether Asd4 interacted with *GLN1* DNA *in vivo*. Using Anti-GFP, we immunoprecipitated chromatin samples from Δ*asd4 ASD4* ^*GFP*^ strains, and from the WT lacking the *ASD4* ^*GFP*^ allele, following growth on 1% GMM with 10 mM NH_4_^+^ as the sole nitrogen source. ChIP-qPCR detected a significant enrichment (*Student’s t-test* p ≤ 0.05) of *GLN1* DNA in ChIP samples from strains expressing Asd4^GFP^ compared to WT (**Fig 3A**). The *GLN1* signal/ input ratio for Asd4^GFP^ ChIP was 9.6-fold higher than for WT ChIP (ie. the background), thus demonstrating a physical interaction between Asd4^GFP^ and *GLN1* DNA that is consistent with the transcriptional data (**Figs 2**, **S2 and S3**). Also, the positioning of the ChIP-qPCR primers used to detect *GLN1* suggests Asd4 binding occurs in the 5 ‘ region of the gene, which might be consistent with the presence of predicted GATA-binding sequences in the promoter region of *GLN1* [23]. Taken together, we conclude that *GLN1* is a cryptic glutamine synthetase-encoding gene normally silenced in WT by Asd4.

To determine if *GLN1* expression in Δ*asd4* mutant strains affected appressorium development, we deleted *GLN1* from the WT and Δ*asd4* genomes using targeted homologous gene replacement. As expected for a gene that is not normally expressed (**S3 Fig**), loss of *GLN1* in WT did not affect colony morphology, sporulation, appressorium formation or pathogenicity (**S4A–S4D Fig**). However, in the Δ*asd4* Δ*gln1* double mutant strain, steady-state intracellular glutamine pools were restored to WT levels when grown on NH_4_^+^-media (**Fig 3B** and **Table 1)**, indicating that inappropriate *GLN1* expression in Δ*asd4* mutant strains affects nitrogen assimilation and/ or distribution into amino acids. Furthermore, Δ*asd4* Δ*gln1* germ tubes were found to develop melanized appressorium on artificial hydrophobic surfaces (**Fig 3C**) at a significantly higher rate (*Student’s t-test* p ≤ 0.05) than Δ*asd4* mutant strains (**Fig 3D**). Thus, Asd4-dependent silencing of *GLN1* might be required for maintaining intracellular glutamine pools at levels optimal for promoting appressorium formation in WT.

*GLN2* expression is also upregulated in Δ*asd4* mutant strains compared to WT (although not to the same extend as *GLN1*; **S2 Fig**) and deleting *GLN2* in Δ*asd4* mutant strains might also affect glutamine levels and appressorium formation. However, despite numerous attempts, we were unable to generate Δ*asd4* Δ*gln2* double mutant strains in this study, perhaps indicating that whereas *GLN2* functions without *GLN1* under a range of developmental conditions (**S3 Fig**), *GLN1* cannot substitute for *GLN2* in Δ*asd4* mutant strains. The relationship between *GLN2* and *ASD4* requires more articulation but does not affect our central conclusion that altering intracellular glutamine levels in Δ*asd4* mutant strains due to *GLN1* expression affects appressorium formation.

### Appressorium formation requires a glutamine-dependent signal

In addition to restoring glutamine levels, deleting *GLN1* in the Δ*asd4* background also restored intracellular pool levels of asparagine and valine (**Table 1**)**.** However, three lines of evidence suggested that the reduction in intracellular glutamine levels (rather than global changes in nitrogen assimilation and distribution) was linked to appressorium formation in the Δ*asd4* Δ*gln1* double mutant compared to the Δ*asd4* parental strain. Firstly, Δ*asd4* Δ*gln1* strains continued to grow poorly on NH_4_^+^- media (**S4E Fig**), and aspartate, alanine and arginine levels were not remediated in the Δ*asd4* Δ*gln1* double mutant strain (**Table 1**). This indicates that nitrogen assimilation and/ or distribution remained defective in Δ*asd4* Δ*gln1* strains but did not prevent appressorium formation. Secondly, treating Δ*asd4* spores with the glutamine synthetase inhibitor L-methionine sulphoximine (MSX), shown in yeast to reduce intracellular glutamine levels [28], significantly improved (*Student’s t-test* p ≤ 0.05) Δ*asd4* appressorium formation rates on artificial surfaces (**Fig 3E**). Thirdly, in yeast [20, 28, 29] and mammals [30], glutamine (amongst other metabolites) acts as a signal to activate the conserved target of rapamycin (TOR) pathway and facilitate growth under nutrient-sufficient conditions. The specific TOR kinase inhibitor rapamycin inactivates TOR and induces starvation-like responses in yeast and mammals [20, 31, 32]. **Fig 3F** shows that treatment of Δ*asd4* spores with rapamycin significantly (*Student’s t-test* p ≤ 0.05) induced appressorium formation on inductive, artificial hydrophobic surfaces compared to untreated controls (**Fig 3F**). Rapamycin treatment also induced appressorium formation in Δ*asd4* mutant strains derived from 70–15 (**S5 Fig**). Intracellular glutamine levels were not affected by treatment with rapamycin and remained elevated in Δ*asd4* mutant strains compared to the Guy11 WT (**Fig 3G**), suggesting rapamycin bypasses the elevated glutamine signal in Δ*asd4* mutant strains to promote appressoria formation. Moreover, whereas WT appressorium formation rates were not affected by rapamycin treatment on inductive hydrophobic surfaces (**Fig 3F**), rapamycin treatment significantly (*Student’s t-test* p ≤ 0.05) enhanced the rate of appressorial formation for WT and Δ*asd4* mutant strains on non-inductive hydrophilic surfaces (glass coverslips) **(Fig 3H)**. Taken together, these results suggest that (i) Asd4-dependent glutamine metabolism and the resulting glutamine pool sizes are important determinants of appressorium formation, and (ii) glutamine signaling might regulate appressorium formation via the TOR signaling pathway.

### Evidence for a functional connection between Asd4 and TOR signaling

**Fig 4:**
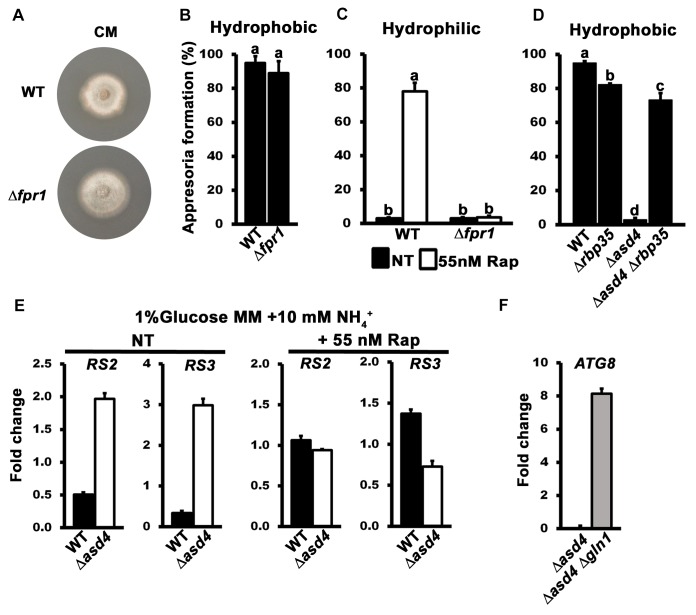
The TOR signaling pathway is misregulated in Δ*asd4* mutant strains. (A) The colony morphology of Δ*fpr1* mutant strains on complete media was not altered compared to WT. Strains were grown for 10 days. (B) Appressorial formation rates of Δ*fpr1* mutant strains were not significantly different (*Student’s t-test* p > 0.5) from WT on hydrophobic artificial surfaces. (C) Treating spore suspensions with 55 nM rapamycin (Rap) did not induce appressorium formation in Δ*fpr1* mutant strains on non-inductive hydrophilic surfaces compared to WT. (D) Appressorial formation rates on artificial hydrophobic surfaces were significantly elevated in an Δ*asd4* Δ*rbp35* double mutant compared to the Δ*asd4* single mutant. (E) The expression of the *M*. *oryzae* ribosomal protein genes *RS2* and *RS3* was increased in *Δasd4* mutant strains compared to WT after growth in 1% GMM with 10 mM NH_4_^+^ for 16h. Treatment with 55 nM Rap restored the expression of *RS2* and *RS3* in Δ*asd4* mutant strains to WT levels. Expression levels were normalized against *TUB2* gene expression and are given as relative fold changes. (F) *ATG8* gene expression is upregulated in *Δasd4 Δgln1* mutant strains compared to the *Δasd4* mutant when normalized against *TUB2* gene expression. (B-D) Error bars are standard deviation. Bars with different letters are significantly different (*Student’s t-test* p ≤ 0.05). Values are the average of the number of appressoria formed at 24 hpi from 50 spores per coverslip, repeated in triplicate. NT = no treatment. (E-F) Values are the mean of three independent replicates. Error bars are SD.

The previous section suggested that Asd4 might modulate intracellular glutamine levels to control appressorium formation via TOR. We next sought more evidence for a functional connection between Asd4 and TOR signaling. Firstly, we intended to confirm that rapamycin could affect appressorium formation by acting directly on TOR, as opposed to having off-target effects on unrelated processes. To achieve this goal, we identified *MoFRP1*, the *M*. *oryzae* homologue of the yeast *FRP1* gene encoding the FK506/rapamycin-binding protein FKBP12. The FKBP-rapamycin complex physically interacts with TOR to inhibit its activity, and TOR is the conserved target of FKBP-rapamycin [20]. However, FKBP12 does not interact with TOR in the absence of rapamycin and consequently in yeast, *FRP1* deletion strains are viable but are not responsive to rapamycin [33]. We generated Δ*fpr1* mutant strains that were indistinguishable from WT on plates (**Fig 4A**) and formed appressoria on hydrophobic surfaces (**Fig 4B**). These results are consistent with previous studies that showed how the *Botrytis cinerea* FKBP12 ortholog is not required for plant pathogenicity [34]. However, rapamycin failed to induce appressorium formation by Δ*fpr1* mutant strains on hydrophilic surfaces (**Fig 4C**). These results demonstrate that rapamycin treatment requires FKBP12 to affect appressorium formation and thus, *a priori*, FKBP-rapamycin must be acting on its conserved target TOR.

Secondly, we sought genetic evidence that TOR inactivation restored appressoria formation in Δ*asd4* mutant strains in order to corroborate our pharmacological data. We hypothesized that disrupting the sole copy of the TOR-encoding gene*TOR1* in Δ*asd4* mutant strains would restore appressorium formation. However, we were unable to generate Δ*tor1* mutant deletion strains in Guy11 or Δ*asd4* strains, likely due to an essential role for the TOR protein in cell viability. This is consistent with studies in *Fusarium graminearum* that were also unable to yield targeted deletions of the single *FgTOR* gene [35]. Future work might involve gene silencing of *TOR1* rather than deletion, but we did not attempt that here, in part because gene silencing in *M*. *oryzae* has not been developed to the stage where targeted genes can be switched off at specific stages of development, and in part because we had an alternative strategy involving the Δ*rbp35* mutant strain. *RBP35* encodes an *M*. *oryzae* RNA-binding protein involved in processing RNA transcripts essential for rice root colonization [36]. Loss of *RBP35* leads to downregulation of the TOR signaling pathway [36, 37]. We hypothesized that deleting *ASD4* in the Δ*rbp35* mutant strain would permit appressorium formation because the downregulation of TOR signaling resulting from the Δ*rbp35* allele would counteract the upregulation of the TOR signaling pathway resulting from intracellular glutamine accumulation in the Δ*asd4* mutant strain. **Fig 4D** shows that, as predicted, the Δ*asd4* Δ*rbp35* double mutant produced significantly more appressoria on inductive, hydrophobic surfaces than the Δ*asd4* single mutant strain. This provides genetic evidence that TOR signaling lies downstream of Asd4 and is activated in the Δ*asd4* mutant strains to prevent appressorium formation.

Finally, we sought to demonstrate that TOR signaling was perturbed in Δ*asd4* strains by analyzing the expression of TOR readout genes in WT and Δ*asd4* mutant strains. *RS2* and *RS3* encode ribosomal proteins that have been shown previously to be elevated in expression when TOR is active but reduced in expression when TOR is inactivated following rapamycin treatment [38]. **Fig 4E** shows that the *RS2* and *RS3* genes were elevated in expression in Δ*asd4* mutant strains following axenic growth compared to WT, and this expression pattern was reversed when rapamycin was added to the growth media. Furthermore, *ATG8* is an autophagy gene whose expression is repressed by active TOR. Autophagy is required for appressorium maturation in *M*. *oryzae* [39], and is a processes inhibited by active TOR in yeast and mammals [40]. **Fig 4F** shows that *ATG8* expression was repressed in Δ*asd4* mutant strains following axenic growth compared to the Δ*asd4* Δ*gln1* double mutant.

When considered together, these results indicate that Asd4 acts upstream of TOR (via glutamine) in order to regulate appressoria formation. Consequently, TOR signaling is perturbed in Δ*asd4* mutant strains.

### TOR activation inhibits appressorium formation downstream of cAMP/ PKA signaling

**Fig 5:**
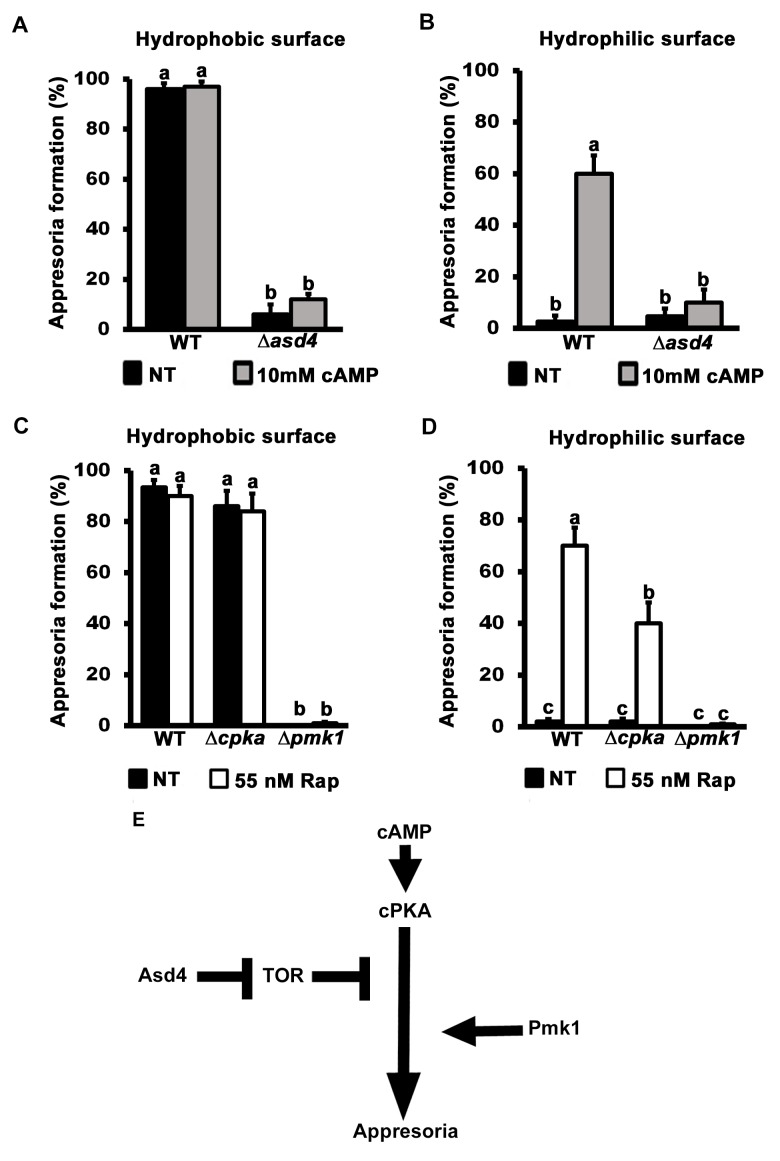
TOR inhibits appressorium formation downstream of cAMP/ PKA signaling. Treating spore suspensions with 10 mM cAMP did not induce appressorium formation by Δ*asd4* mutant strains on hydrophobic (A) or (unlike WT) non-inductive hydrophilic surfaces (B). Treatment with 55 nM rapamycin (C and D) induced appressorium formation by Δ*cpka* mutant strains on non-inductive hydrophilic surfaces but did not induce appressorium formation by Δ*pmk1* mutant strains on any surface. (A-D) Mean appressorial formation rates were determined at 24 hpi from 50 spores per hydrophilic slide or hydrophobic coverslip, repeated in triplicate. NT = no treatment. Error bars are the standard deviation. Bars with different letters are significantly different (*Student’s t-test* p ≤ 0.05). (E) Appressorium formation requires functional cAMP/ PKA- and MAP kinase-signaling pathways, and Asd4-dependent inactivation of TOR downstream of cPKA.

A previously unknown outcome of this work is the discovery that rapamycin treatment can generate appressoria on non-inductive hydrophilic surfaces (**Fig 3H**). cAMP treatment, or mutations that constitutively activate cAMP/ PKA signaling, also enable appressoria to form on non-inductive hydrophilic surfaces [8]. We next asked if a relationship existed between TOR and the cAMP/ PKA- and MAP kinase-signaling pathways by first treating Δ*asd4* spores with cAMP. cAMP treatment did not restore appressorium formation to Δ*asd4* mutant strains on either inductive hydrophobic (**Fig 5A**) or, in contrast to WT, on non-inductive hydrophilic surfaces (**Fig 5B**). This indicates that activated TOR in Δ*asd4* mutant strains blocks appressorium formation downstream of cPKA. However, although downstream of cAMP/ PKA signaling, Asd4 is not under direct cPKA control because if so, cPKA would be required for Asd4 function and Δ*cpka* strains would be expected to phenocopy Δ*asd4* strains. However, **S6A Fig** shows that Δ*cpka* mutant strains grew better than Δ*asd4* strains on NH_4_^+^ media. Thus, *CPKA* is not likely epistatic to *ASD4*.

Further evidence that TOR acts downstream of cPKA is shown in **Fig 5C and 5D**. Spores of the cAMP/ PKA signaling mutant Δ*cpka* and the MAP kinase mutant Δ*pmk1* were treated with rapamycin. In common with previous reports [15], by 24 hpi, Δ*cpka* mutant strains had formed appressoria on inductive hydrophobic surfaces at the same rate as WT (**Fig 5C**). On non-inductive hydrophilic surfaces, Δ*cpka* spores treated with rapamycin formed significantly more (*Student’s t-test* p ≤ 0.05) appressoria than untreated spores (**Fig 5D**). In contrast, appressorium formation by Δ*pmk1* strains was not induced by rapamycin treatment on hydrophobic (**Fig 5C**) or hydrophilic (**Fig 5D**) surfaces. Thus, inactivating TOR promotes appressorium formation in a cPKA-independent, Pmk1-dependent manner.

Treatment with cAMP results in germ tube tip differentiation in Δ*pmk1* strains [16] resulting in hooking and swelling but not appressorium formation **S6B Fig**). This places Pmk1 function downstream of cAMP/ PKA [16]. In contrast, Δ*asd4* mutant strains treated with 10 mM cAMP on hydrophilic surfaces did not exhibit hooking or swelling and the germ tube tips remained undifferentiated (**S6B Fig**). This places Asd4 function upstream of Pmk1.

Together, these results are consistent with the model shown in **Fig 5E** which shows that appressorium formation requires both activation of the cAMP/ PKA and MAP kinase signaling pathways and inactivation of the TOR signaling pathway, the latter via Asd4-dependent glutamine metabolism. Conversely, activated TOR in Δ*asd4* strains inhibits appressorium formation downstream of cAMP/ PKA but upstream of Pmk1 (**Fig 5E**).

### Δ*asd4* appressoria resulting from rapamycin treatment or *GLN1* deletion were not infection-competent

**Fig 6:**
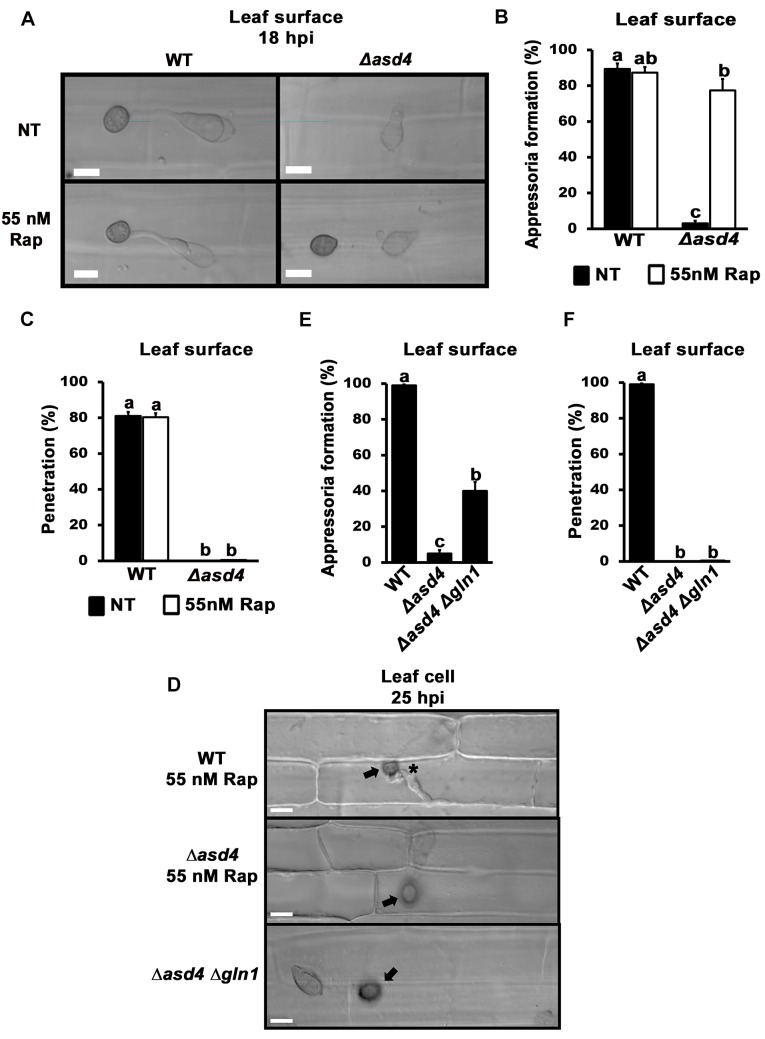
Δ*asd4* mutant strains treated with rapamycin or lacking *GLN1* formed melanized appressoria on rice leaf surfaces that were not infection-competent. Rapamycin treated spores of Δ*asd4* mutant strains formed melanized appressoria at 18 hpi on detached leaf sheath surfaces of the susceptible rice cultivar CO-39 (A) at rates that were not different to WT (B). However, the resulting Δ*asd4* appressoria were non-functional and had not penetrated the leaf cuticle (C), or (D) produced primary hyphae in the rice cell (indicated by the asterisk for WT), by 25 hpi. Arrows indicate appressoria on the surface of the leaf. (E) Like rapamycin treatment, deleting *GLN1* in Δ*asd4* mutant strains also promoted appressorium formation on detached leaf surfaces by 18 hpi, but these Δ*asd4* Δ*gln1* appressoria were non-functional and, unlike WT, had not penetrated the leaf surface by 25 hpi (D, F). (B, E) Values are the average number of appressoria formed from 50 spores per rice leaf sheath, repeated in triplicate. (C, F) Mean penetration rates were calculated from 50 appressoria per leaf sheath, repeated in triplicate. Error bars are the standard deviation. Bars with different letters are significantly different (*Student’s t-test* p ≤ 0.05).

We next sought to determine the physiological relevance of the connection between Asd4 and TOR under infection conditions. Δ*asd4* spores that had been treated with rapamycin and applied to detached rice leaf sheath surfaces formed melanized appressoria (**Fig 6A**) at rates that were not significantly different to rapamycin treated WT spores (p = 0.08; **Fig 6B**). However, the resulting Δ*asd4* appressoria were non-functional and unable to penetrate rice leaf surfaces (**Fig 6C and 6D**). Similarly, untreated spores of the Δ*asd4* Δ*gln1* double mutant, compared to WT and the Δ*asd4* parental strain, formed appressoria on leaf sheaths (**Fig 6E**), but none were observed penetrating the host leaf surface (**Fig 6D and 6F**). These results provide evidence that, on the one hand, Asd4-dependent TOR inactivation is required for appressorium formation during rice infection. On the other hand, Asd4 is shown here to have roles in the pre-penetration stage of infection that might be independent of TOR and which are currently unknown.

## Discussion

New insights into the molecular pathways that regulate plant invasion by pests could reveal attractive targets for effectively managing a range of diseases. Many fungal pathogens rely on appressoria to infect host cells [2], and appressorial developmental, at least in *M*. *oryzae*, is dependent on positive-acting cAMP/ PKA- and MAP kinase-signaling pathways [5, 10]. Here, we undertook the first steps in providing a mechanistic account of a negative-acting regulator of appressorium formation in *M*. *oryzae*. Appressoria form under nutrient-free, hydrophobic conditions, and we showed here that an activated TOR signaling pathway blocks this process. TOR signaling was found to be constitutively active in the GATA factor mutant strain Δ*asd4*, and characterizing Asd4 function provided several unique insights into the biology of infection-related development. Our results are consistent with a model whereby Asd4 represses the expression of a glutamine synthetase orthologue, *GLN1*, and down-regulates the expression of other structural genes involved in nitrogen assimilation and glutamine turnover. We propose this maintains intracellular glutamine pools at levels that are not sufficient to activate TOR. Perturbing glutaminolysis and activating *GLN1* expression in Δ*asd4* mutant strains affected the intracellular steady-state pools of glutamine and activated TOR, resulting in inhibition of the cAMP/ PKA signaling pathway and loss of appressorium formation. Inactivating TOR restored appressorium formation by Δ*asd4* mutant strains. This was most evident on host leaf surfaces where Δ*asd4* appressoria formed at rates indistinguishable from WT after rapamycin treatment. When taken together, the key novel features of the work described here include elucidating a role for TOR in inhibiting appressorial formation; discovering TOR inactivation requires Asd4; and identifying TOR as a regulator of cAMP/ PKA signaling downstream of cPKA but upstream of its connection with the MAP kinase pathway.

In yeast, the GATA transcription factors Gln3 and Gat1 are required for utilizing non-preferred nitrogen sources and are downstream targets of TOR [20, 41]. TOR is activated in response to carbon and nitrogen sufficiency cues, including glutamine [20, 28, 29, 42], leading to the induction of anabolic processes and growth. Under these conditions, Gln3 and Gat1 are maintained in the cytoplasm. TOR inactivation due to nutrient limitation results in increased autophagy, reduced protein synthesis and increased nitrogen catabolic gene expression following Gln3 and Gat1 nuclear localization [20, 28, 29, 43]. Thus, TOR controls Gln3 and Gat1 activity in yeast. In addition to the Gln3 and Gat1 transcriptional activators, Dal80 and Gzf3 are yeast GATA factors that, like Asd4, act as transcriptional repressors [42]. However, in contrast to the situation described here (whereby Asd4 is upstream of TOR and mediates its activity by controlling glutamine metabolism), no comparable roles in controlling TOR signaling have been described for the yeast Dal80 and Gzf3 GATA factors [20, 44]. Moreover, GATA factors were not found in a recent screen of yeast genes necessary for TOR inactivation [44]. Thus, Asd4 is revealed here as a novel TOR regulator, but whether this role is conserved in other fungi, or necessitated in *M*. *oryzae* due to specific demands for TOR-related processes during the infection cycle, is not known.

This work has provided new mechanistic insights into the control of TOR during the *M*. *oryzae*-rice interaction. In general, though, the role of TOR in phytopathology is not well understood, although nonselective macroautophagy- an output of inactive TOR signaling in yeast—is necessary for the maturation of incipient appressoria in *M*. *oryzae* [45]. Evidence of a role for TOR in root colonization by *M*. *oryzae* has also been presented [36] whereby TOR signaling is downregulated in the non-pathogenic RNA processing mutant Δ*rbp35*. In the wheat pathogen *F*. *graminearum*, loss of *FgFKBP12* and mutations in *FgTOR1* abolished the toxicity of rapamycin, while downstream components of the TOR pathway—FgSit4, FgPpg and FgTip41—were shown to have roles in virulence, development and mycotoxin production [35]. A separate study has characterized the serine/threonine-protein kinase *SCH9*, an important downstream target of yeast TORC1, in *F*. *graminearum* and *M*. *oryzae* [46]. Δ*Fgsch9* deletions strains were impaired for conidiation, mycotoxin production and virulence on wheat heads, and produced smaller spores than the *F*. *graminearum* parental strain. Δ*Mosch9* mutant strains exhibited reduced conidia and appressorial sizes than WT and were defective, though not abolished, in plant infection [46]. Recently, we have shown how the biotrophic growth of *M*. *oryzae* in rice cells requires a transketolase—dependent metabolic checkpoint involving the activation of TOR [38]. Loss of transketolase function resulted in Δ*tkl1* mutant strains that formed functional appressoria, penetrated the rice cuticle and elaborated invasive hyphae. However, Δ*tkl1* strains were depleted for ATP, an agonist of TOR, and these strains underwent mitotic delay and reduced hyphal growth in rice cells due to the inactivation of TOR [38]. How TOR controls the cell cycle during *M*. *oryzae* biotrophy is not known. Thus, extending these observations on the roles of TOR signaling during plant pathogenesis, and integrating them into testable models of phytopathogen growth and development, will be a future challenge.

Our results presented here indicate how nitrogen turnover is an important feature of appressorial morphogenesis. Because appressoria develop on the nutrient-free surface of the leaf, internal nitrogen sources that contribute to the glutamine pools affecting TOR must be generated from the recycling of endogenous proteins during autophagic cell death of the spore. This would be consistent with previous work that determined ubiquitin-mediated proteolysis was required for many aspects of *M*. *oryzae* development, including appressorium function [47]. How protein turnover during appressorium formation integrates with Asd4-dependent nitrogen assimilation, glutaminolysis and TOR activity is therefore an important question for rice blast research that could shed light on the relationships between GATA and TOR in different systems.

Downstream of Asd4, how the TOR pathway intersects and inhibits the cAMP/ PKA signaling pathway is not known. In yeast, controversy has developed regarding how TOR and PKA signaling pathways regulate common protein targets, with some models suggesting the pathways act in parallel, and some models suggesting TOR is upstream of PKA [48]. Both models are likely valid because recent work has shown that the regulatory subunit of PKA can be a direct target of TOR in yeast, although PKA phosphorylation by TOR occurs selectively and is not global [48]. Our study of *M*. *oryzae* places TOR downstream and inhibitory to cAMP/ PKA signaling during appressorium formation and thus provides an opportunity to uncover new relationships between these two fundamental pathways. This would include identifying common targets that control appressorium formation. One point of shared control for the two pathways could be autophagy because Δ*cpka* and Δ*pmk1* mutants are unable to undergo autophagy in *M*. *oryzae* [39], and PKA is necessary for autophagy in yeast [49]. Thus, focusing on TOR, PKA and autophagy will likely yield important insights into appressorial biology.

Another important area of future study will be uncovering the role of Asd4 in cuticle penetration. Although Δ*asd4* Δ*gln1* strains and Δ*asd4* spores treated with rapamycin could form melanized, mature appressoria, they were unable to form penetration pegs. This suggests Asd4 might play a TOR-independent role in the late stages of appressorium maturation and/ or the regulation of penetration peg formation. Successful peg penetration is dependent on the Pmk1 MAP kinase target Mst12 [9, 50], and on NADPH oxidase-dependent control of septin and F-actin reorganization [51]. Also, in addition to the Pmk1 MAP kinase pathway that is essential for appressorium formation, another MAP kinase pathway in *M*. *oryzae*, involving Mps1, is not involved in appressorium formation but is required for penetration [52]. Determining if and how Asd4 intersects with these processes will likely yield important new discoveries about appressorium function.

In summary, this work demonstrates the utility of performing axenic physiological analyses to make testable inferences regarding the metabolic strategies underlying *M*. *oryzae* infection of host plants. This has revealed that TOR inactivation requires Asd4, and that the TOR pathway is a previously unknown negative-acting regulator of cAMP/ PKA signaling. The results presented here thus provide mechanistic insights that extend our basic knowledge of regulatory networks in fungi by revealing novel connections between GATA-, TOR- and PKA-mediated signaling within the context of appressorium morphogenesis. Given the wealth of knowledge about the role of TOR in yeast physiology and human pathologies, further explorations of the function of TOR in *M*. *oryzae* and other appressorium-forming phytopathogens could provide new tools and avenues for alleviating the global burden of plant diseases attributable to fungi. Conversely, revealing Asd4 as a new TOR regulator might shed light on aspects of developmental control that could be applicable to a wide range of cellular processes across taxa.

## Materials and Methods

### Strains and physiological tests

The strains used in this study are listed in Table 2. Strains were grown on complete medium (CM) containing 1% (W/V) glucose, 0.2% (W/V) peptone, 0.1% (W/V) yeast extract and 0.1% (W/V) casamino acids, or on minimal medium (MM) containing 1% glucose and 0.6% sodium nitrate, unless otherwise stated, as described in [18]. For the growth tests, nitrogen sources were used in MM at 10 mM concentrations, unless otherwise specified. Plate images were taken with a Sony Cyber-shot digital camera, 14.1 mega pixels. For spore counts, 10 mm^2^ blocks of mycelium were transferred to the center of each plate, and the strains grown for 12 days at 26 °C with 12 hr light/dark cycles. Spores were harvested in sterile distilled water, vortexed vigorously and counted on a haemocytometer (Corning). Spores were counted independently at least three times.

**Table 2:** *Magnaporthe oryzae* strains used in this study.

Strains	Genotype	Reference
Guy11	*M*. *oryzae* wild type isolate (WT) used throughout this study unless otherwise specified.	[18]
Δ*asd4*	GATA factor (MGG_06050) deletion mutant of Guy11 and used throughout this study unless otherwise specified.	[18]
Δ*asd4 ASD4* ^*GFP*^	Asd4 complementation strain expressing the Asd4::GFP fusion protein under its native promoter in the Δ*asd4* mutant background.	*This study*
Δ*gln1*	Glutamine synthetase (MGG_06888) deletion mutant of Guy11.	*This study*
Δ*asd4* Δ*gln1*	Glutamine synthetase (MGG_06888) deletion mutant of Δ*asd4*.	*This study*
Δ*pmk1*	Map-kinase deletion mutant of Guy11.	[16]
Δ*cpka*	cAMP dependent kinase A deletion mutant of Guy11.	[15]
Δ*fpr1*	FKBP12 (MGG_06035) deletion mutant of Guy11.	*This study*
Δ*rbp35*	RNA-binding protein (MGG_02741) deletion mutant of Guy11.	[36]
Δ*asd4* Δ*rbp35*	GATA factor (MGG_06050) deletion mutant in the Δ*rbp35* mutant background.	*This study*
70–15	*M*. *oryzae* wild type isolate.	[23]
Δ*asd4* [70–15]	GATA factor (MGG_06050) deletion mutant of 70–15.	*This study*

The spore suspensions were adjusted to a concentration of 1 x 10^5^ spores/ml to perform the appressoria formation tests. Rapamycin (Rap; LC Laboratories, USA) and monobutyryl cyclic AMP (cAMP; Sigma-Aldritch, USA) were added to the spore suspensions at a concentration of 55 nM and 10 mM, respectively. Appressorial development was evaluated on inductive, hydrophobic plastic coverslips and non-inductive, hydrophilic glass slides. Both substrates were inoculated with 200 μl of each spore suspension and appressoria were observed 24 hrs post-inoculation (hpi). Rates were determined by counting the number of appressoria formed from 50 conidia per coverslip, repeated in triplicate for each strain [53]. Concentrations of 55 nM—1 μM rapamycin could induce appressorial formation of WT on hydrophilic surfaces.

### Plant infection assays and live-cell imaging

Rice plant infections were made using a susceptible rice (*Oryza sativa*) cultivar, CO-39, as described previously [18]. Fungal spores were isolated from 12–14 day-old plate cultures and spray-inoculated onto rice plants of cultivar CO-39 in 0.2% gelatin at a concentration of 1 x 10^5^ spores/ml, and disease symptoms were allowed to develop under conditions of high relative humidity for 120 hrs.

Live-cell imaging was performed as described previously [53] using 3 cm-long leaf sheath segments from 3–4 week-old rice plants and injecting one end of the sheath with a spore suspension of 1 x 10^5^ spores/ml in 0.2% gelatin. At the time points indicated, leaf sheaths were trimmed and observed using a Nikon Eclipse 50i microscope and a Nikon D100 digital net camera. The average rates of appressorium formation and penetration were determined for each strain, in triplicate, by analyzing 50 spores or appressoria per rice cuticle [53].

Asd4^GFP^ was imaged as described previously for H1:RFP [38] using 488 nm and 500–550 nm for excitation and emission wavelengths, respectively.

### Genetic manipulations

Gene functional analysis was achieved by the split marker method described in [18], using the oligonucleotide primers shown in S4 Table. *GLN1* was replaced in the *Δasd4* parental strain using the hygromycin B resistance selectable marker, *hph*. *MoFPR1* and *GLN1* were replaced in the Guy11 genome using the *ILV1* gene conferring resistance to sulphonyl urea [18]. *ASD4* was deleted from the 70–15 background using *ILV1*. *ASD4* was deleted in the *Δrbp35* parental strain using the *Bar* gene conferring bialaphos resistance [18]. Gene deletions were verified by PCR as described previously [18]. The original *Δasd4* mutant strain [18] was complemented with *ASD4* ^GFP^ under its native promoter, constructed using the vector pDL2 and the primers ASD4-G F/R (S4 Table), following the protocol of Zhou et al. [54].

### Gene transcript analysis

Strains were grown for 48 h in CM before switching to minimal media for 3 h and 16 h, as indicated. For *in planta* expression studies, detached rice leaf sheaths were inoculated with WT and harvested at the indicated timepoints. Mycelia and leaves were frozen in liquid nitrogen, and lyophilized for 36 hrs. RNA was extracted from fungal mycelium using the RNeasy mini kit from Qiagen. RNA was converted to cDNA using the qScript reagents from Quantas. Real time quantitative PCR (qPCR) was performed on an Eppendorf Mastercycler Realplex using the recommended reagents with primers designed using the netprimer software program (S4 Table). qPCR data was analyzed using the Realplex software. Thermocycler conditions were: 10 min at 95°C, followed by 40 cycles of 95°C for 30 sec, 63°C for 30 sec and 72°C for 30 sec.

### Chromatin immunoprecipitation (ChIP)

ChIP was performed as described in [55]. WT and *Δasd4 ASD4* ^GFP^ complementation strains were grown in liquid CM for 48 h before switching to 1% GMM with 10 mM NH_4_^+^ for 16 h. Three biological replicates were performed per strain. Thirty per cent of each DNA aliquot was saved prior to ChIP and served as the input controls. Anti-GFP mAB-Agarose (D125-8, MBL) was used to precipitate Asd4^GFP^-bound chromatin. A control ChIP was run in parallel using Mouse IgG-Agarose (A0919, Sigma). The quantification of input and precipitated *GLN1* DNA was performed at least in triplicate using qPCR and the specific *GLN1* primers shown in S4 Table. *GLN1* DNA enrichment by Asd4^GFP^ ChIP was confirmed by calculating the values of *GLN1* DNA obtained following Anti-GFP immunoprecipitation (the signal) relative to the levels of *GLN1* in the input controls, then comparing the *GLN1* signal-to-input ratio derived from the *Δasd4 ASD4* ^GFP^ samples against those of the WT negative control lacking Asd4^GFP^.

### Amino acid quantification

Amino acid analysis was performed by LC-MS/MS using the aTRAQ kit provided by ABSciex (Framingham, MA). Samples of lyophilized ground mycelia were first washed with water by suspension and centrifugation at 4 ^o^C. The supernatants were aspirated and the pellets were used for extraction. The extractions were performed using 90% MeOH with 128 μM Norleucine added as internal standard. After incubation at -55 ^o^C, a 10 μL aliquot was removed and concentrated by SpeedVac Centrifugation followed by derivatization according to the aTRAQ protocol. The parameters for the MRM acquisition, chromatography and ion source operation were also according to the aTRAQ protocol (Curtain gas = 20, CAD = Medium, IS = 1500; TEM = 600, GS1 = GS2 = 60, heater on) employing a Nova Pak C-18 4 μm 3.9x150mm from Waters Corp. (Milford, MA) for the separation of the tagged amino acids with a sample injection volume of 2 μL. Amino acid concentrations were calculated from the ratio of areas (Heavy aTRAQ/light aTRAQ labeled standards) and corrected for losses for the entire procedure by means of the Nle Internal Standard area recovery.

## Supporting Information

S1 Fig *ASD4* is essential for appressorium formation in Guy11 and 70–15 parental strains. (A) Deleting *ASD4* from the genome of the wild type isolate 70–15, like deleting *ASD4* in the Guy11 background, resulted in Δ*asd4* (70–15) mutant strains that were reduced in radial growth after 10 days on GMM with 10 mM NH_4_^+^ compared to the 70–15 parental strain. Error bars are standard deviation. Bars with different letters are significantly different (*Student’s t-test* p ≤ 0.05). (B) Conidia of the parental strain 70–15 and the Δ*asd4* (70–15) mutant strain were applied to artificial hydrophobic surfaces. At 24 hpi, spores of 70–15 had germinated and formed melanized appressoria at the germ-tube tips (*red arrow*). In contrast, Δ*asd4* mutant spores (like those of Δ*asd4* in the Guy11 background) had germinated but failed to develop appressoria by 24 hpi. (C) Complementing the Δ*asd4* mutant strain derived from Guy11 with a copy of *ASD4* fused to *GFP* and expressed under its native promoter resulted in Δ*asd4 ASD4* ^*GFP*^ complementation strains that were restored for appressoria formation (*red arrows*) on artificial hydrophobic surfaces at 24 hpi. (D) The GATA transcription factor Asd4 fused to GFP localizes to the nucleus during appressoria development on artificial hydrophobic surfaces. Scale bar is 10 μm.
(TIF)

S2 Fig *ASD4* regulates the expression of genes involved in nitrogen assimilation. *GLN1*, *GLN2*, *GDH1*, *GLT1*, and *MGD1* gene expression was analyzed in strains of WT and *Δasd4* after 3 h and 16 h growth in 1% (w/v) glucose MM (GMM) with 10 mM NH_4_^+^ as the sole nitrogen source. Results were normalized against the expression of the β-tubulin gene *TUB2*. Values are the average of three results from at least two independent biological replicates. Error bars are standard deviation.
(TIF)

S3 Fig *GLN1* is not expressed in WT under the conditions studied. (A) Mining the genome-wide transcriptional profiling data generated by the Talbot group using RNAseq and High-Throughput SuperSage analysis [27]—available at http://cogeme.ex.ac.uk/supersage/ - reveals how *GLN2*, *GLT1* and *MGD1* were expressed during appressorium development but *GLN1* and *GDH1* expression was not detectable. (B) *In planta* quantitative real-time PCR (qPCR) analysis of gene expression, using cDNAs obtained from rice leaf sheaths of the susceptible rice cultivar CO-39 inoculated with WT, shows *GLN1* expression was not detected during *M*. *oryzae* infection. Leaf sheaths were inoculated with 1 x 10^5^ spores mL^-1^. RNA was extracted for cDNA synthesis and gene expression analysis at the indicated time points. Results were normalized against the expression of the *M*. *oryzae* actin-encoding gene *MoACT1* and are the average of three independent replications. Error bars are standard deviation. Hpi = hours post inoculation. (C) *GLN1* gene expression was analyzed in WT and *Δasd4* mutant strains after 16 h growth in 1% (w/v) glucose MM (GMM) containing the indicated concentrations of sole nitrogen sources. *GLN1* expression following growth in MM containing glutamine as a sole carbon and nitrogen source was also examined. Gene expression results were normalized against expression of the β-tubulin gene (*β-TUB2*). Values are the average of three replicates. Error bars are standard deviation.
(TIF)

S4 Fig Characterizing *GLN1* function in WT and Δ*asd4* strains. (A) Disrupting *GLN1* in Guy11 does not affect colony morphology on complete media. Strains were grown for 10 days. (B) Δ*gln1* mutant strains were not affected in sporulation after 12 days growth on complete media. (C) Δ*gln1* mutant strains formed appressoria at the same rate as WT on hydrophobic artificial surface. Values are the average of the number of appressoria formed at 24 hpi from 50 spores per coverslip, repeated in triplicate. (D) Δ*gln1* mutant strains were fully pathogenic. Strains were inoculated onto rice (CO-39) at a rate of 1x10^5^ spores mL^-1^. (E) *Δasd4* and *Δasd4 Δgln1* mutant strains were reduced in radial growth compared to WT after 10 days on GMM with 10 mM NH_4_^+^. (B-C) Error bars are standard deviation. Bars with different letters are significantly different (*Student’s t-test* p ≤ 0.05).
(TIF)

S5 Fig Δ*asd4* (70–15) mutant strains were restored for appressoria formation following treatment with Rapamycin. *Δasd4* mutant strains in the 70–15 parental strain background formed appressoria at 24 hpi on artificial hydrophobic surfaces (coverlips) following treatment with 55 nM rapamycin. Bars with different letters are significantly different (*Student’s t-test* p ≤ 0.05). Values are the average of the number of appressoria formed at 24 hpi from 50 spores per coverslip, repeated in triplicate. NT = no treatment.
(TIF)

S6 Fig Asd4 functions downstream of cAMP/ PKA signaling and upstream of MAP kinase signaling. (A) Asd4 acts downstream but independently of cAMP/ PKA signaling. Unlike Δ*asd4*, Δ*cpka* mutant strains were not significantly reduced in growth on GMM with 10 mM NH_4_^+^, indicating Asd4 is not under cAMP/ PKA signaling control. (B) Asd4 regulates appressorium formation upstream of Pmk1. Treatment with 10 mM cAMP resulted in appressorium formation by WT on hydrophilic surfaces. Δ*pmk1* strains responded to cAMP by differentiating germ tube tips on hydrophilic surfaces that hooked and swelled but did not form appressoria, indicating Pmk1 functions downstream of cAMP/PKA signaling [16]. Δ*asd4* mutant strains did not respond to cAMP treatment on hydrophilic surfaces and their germ tube tips did not differentiate, indicating Asd4 functions upstream of Pmk1. Appressoria are denoted by *black arrow*. *Red arrow* denotes differentiated germ tube tips that do not progress beyond hooking and swelling. Bar is 10 μm. Images were made at 24 hpi. NT = no treatment.
(TIF)

S1 Table Colony size (in mm) of *Δasd4* mutant strains compared to Guy11 (WT) following ten days growth on defined minimal media with 1% (w/v) glucose as the sole carbon source and the indicated final concentrations of sole nitrogen sources. (DOCX)

S2 Table Colony size (in mm) of *Δasd4* mutant strains compared to Guy11 (WT) following ten days growth on defined minimal media with the indicated final concentrations of sole carbon and nitrogen sources. (DOCX)

S3 Table Description of *Magnaporthe oryzae* genes analyzed by quantitative RT-PCR in this study. (DOCX)

S4 Table Oligonucleotide primers used in this study. (DOC)
