# Supplementary figures and images for: GATA-Dependent Glutaminolysis Drives Appressorium Formation in Magnaporthe oryzae by Suppressing TOR Inhibition of cAMP/PKA Signaling
Source: PLoS Pathog. 2015 Apr 22;11(4):e1004851. doi: 10.1371/journal.ppat.1004851 (PMC4406744; doi:10.1371/journal.ppat.1004851)

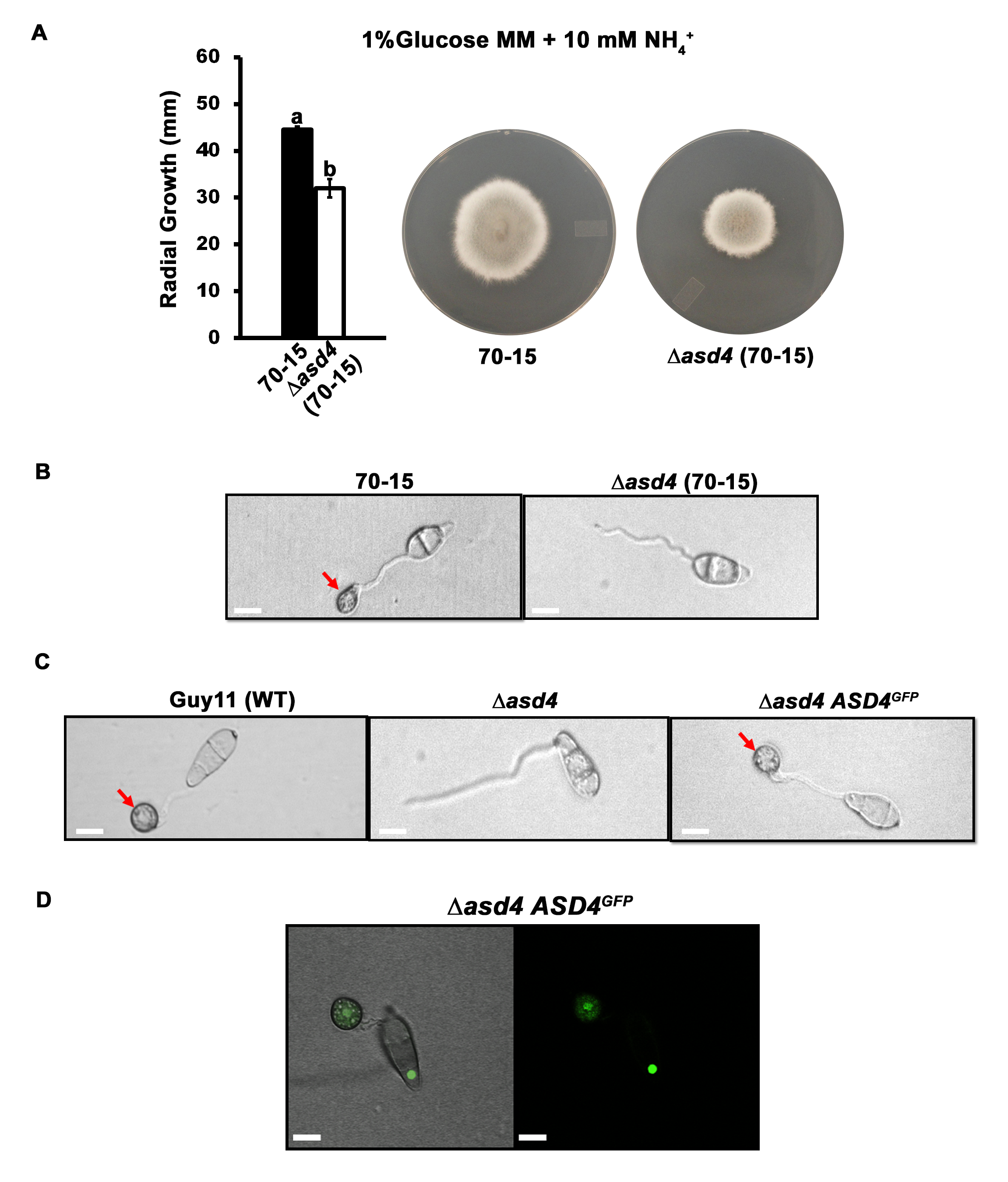

Supplement: S1 Fig — (A) Deleting ASD4 from the genome of the wild type isolate 70–15, like deleting ASD4 in the Guy11 background, resulted in Δasd4 (70–15) mutant strains that were reduced in radial growth after 10 days on GMM with 10 mM NH4 + compared to the 70–15 parental strain. Error bars are standard deviation. Bars with different letters are significantly different (Student’s t-test p ≤ 0.05). (B) Conidia of the parental strain 70–15 and the Δasd4 (70–15) mutant strain were applied to artificial hydrophobic surfaces. At 24 hpi, spores of 70–15 had germinated and formed melanized appressoria at the germ-tube tips (red arrow). In contrast, Δasd4 mutant spores (like those of Δasd4 in the Guy11 background) had germinated but failed to develop appressoria by 24 hpi. (C) Complementing the Δasd4 mutant strain derived from Guy11 with a copy of ASD4 fused to GFP and expressed under its native promoter resulted in Δasd4 ASD4 GFP complementation strains that were restored for appressoria formation (red arrows) on artificial hydrophobic surfaces at 24 hpi. (D) The GATA transcription factor Asd4 fused to GFP localizes to the nucleus during appressoria development on artificial hydrophobic surfaces. Scale bar is 10 μm. (TIF) [file ppat.1004851.s001.tif]

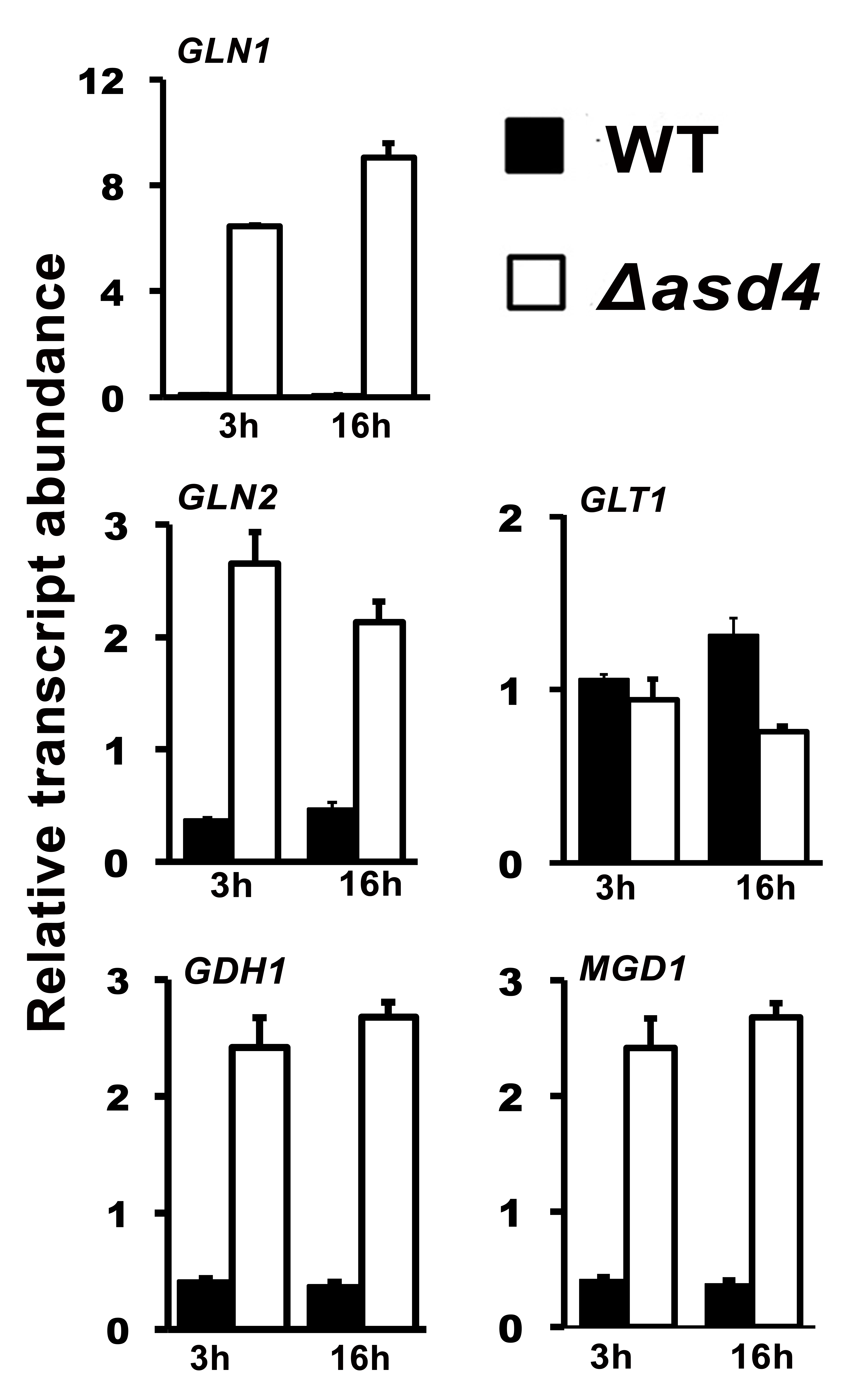

Supplement: S2 Fig — GLN1, GLN2, GDH1, GLT1, and MGD1 gene expression was analyzed in strains of WT and Δasd4 after 3 h and 16 h growth in 1% (w/v) glucose MM (GMM) with 10 mM NH4 + as the sole nitrogen source. Results were normalized against the expression of the β-tubulin gene TUB2. Values are the average of three results from at least two independent biological replicates. Error bars are standard deviation. (TIF) [file ppat.1004851.s002.tif]

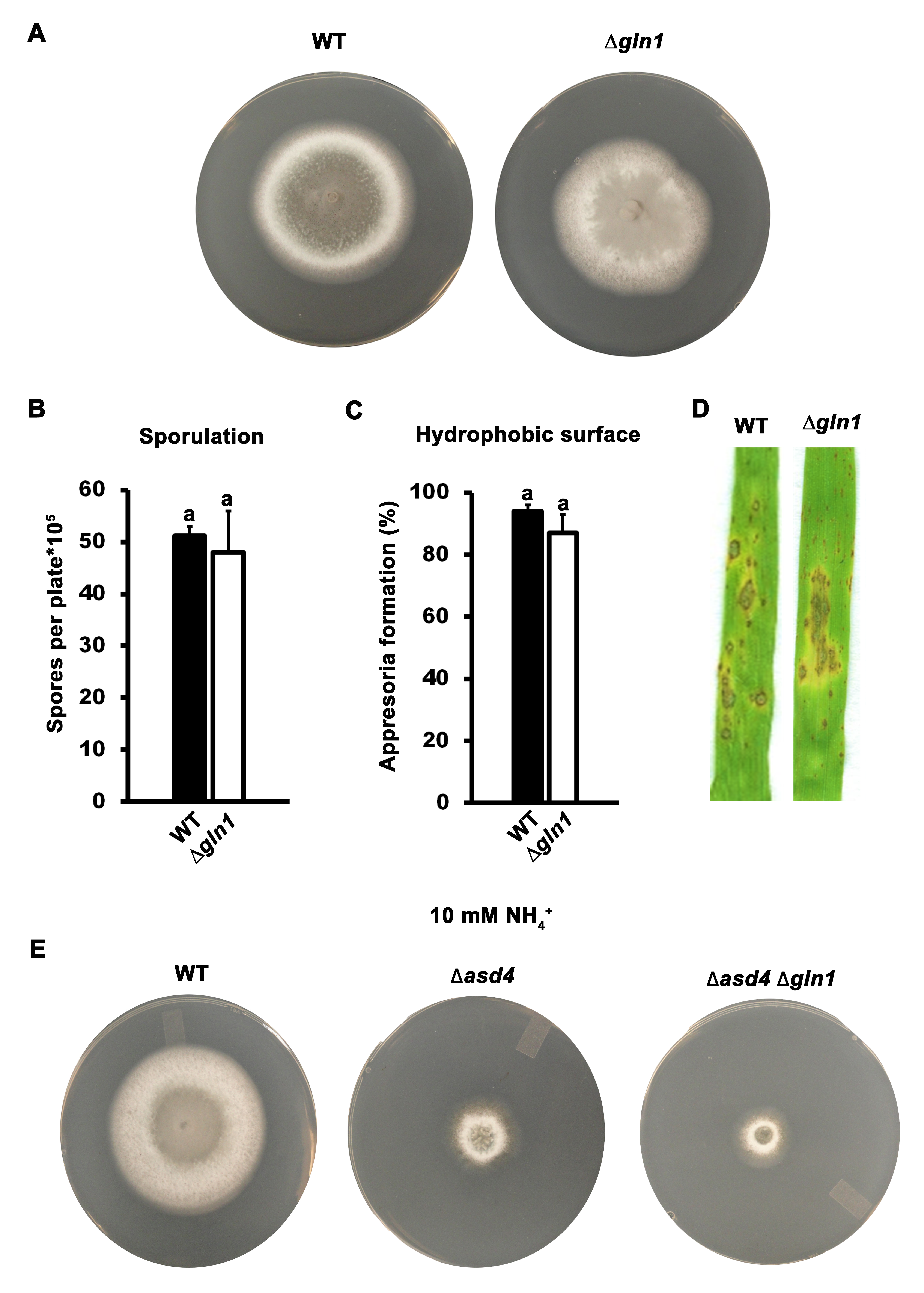

Supplement: S4 Fig — (A) Disrupting GLN1 in Guy11 does not affect colony morphology on complete media. Strains were grown for 10 days. (B) Δgln1 mutant strains were not affected in sporulation after 12 days growth on complete media. (C) Δgln1 mutant strains formed appressoria at the same rate as WT on hydrophobic artificial surface. Values are the average of the number of appressoria formed at 24 hpi from 50 spores per coverslip, repeated in triplicate. (D) Δgln1 mutant strains were fully pathogenic. Strains were inoculated onto rice (CO-39) at a rate of 1x10^5^ spores mL-1. (E) Δasd4 and Δasd4 Δgln1 mutant strains were reduced in radial growth compared to WT after 10 days on GMM with 10 mM NH4 +. (B-C) Error bars are standard deviation. Bars with different letters are significantly different (Student’s t-test p ≤ 0.05). (TIF) [file ppat.1004851.s004.tif]

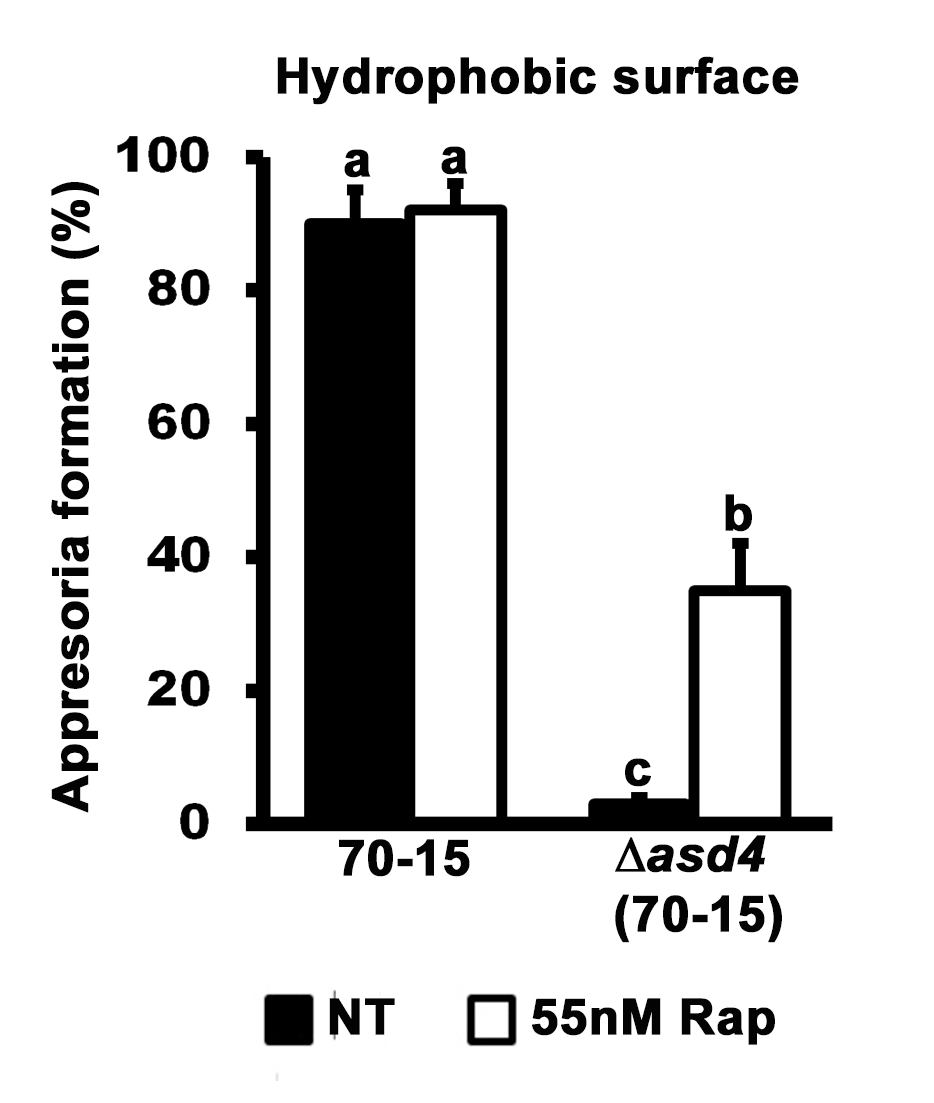

Supplement: S5 Fig — Δasd4 mutant strains in the 70–15 parental strain background formed appressoria at 24 hpi on artificial hydrophobic surfaces (coverlips) following treatment with 55 nM rapamycin. Bars with different letters are significantly different (Student’s t-test p ≤ 0.05). Values are the average of the number of appressoria formed at 24 hpi from 50 spores per coverslip, repeated in triplicate. NT = no treatment. (TIF) [file ppat.1004851.s005.tif]

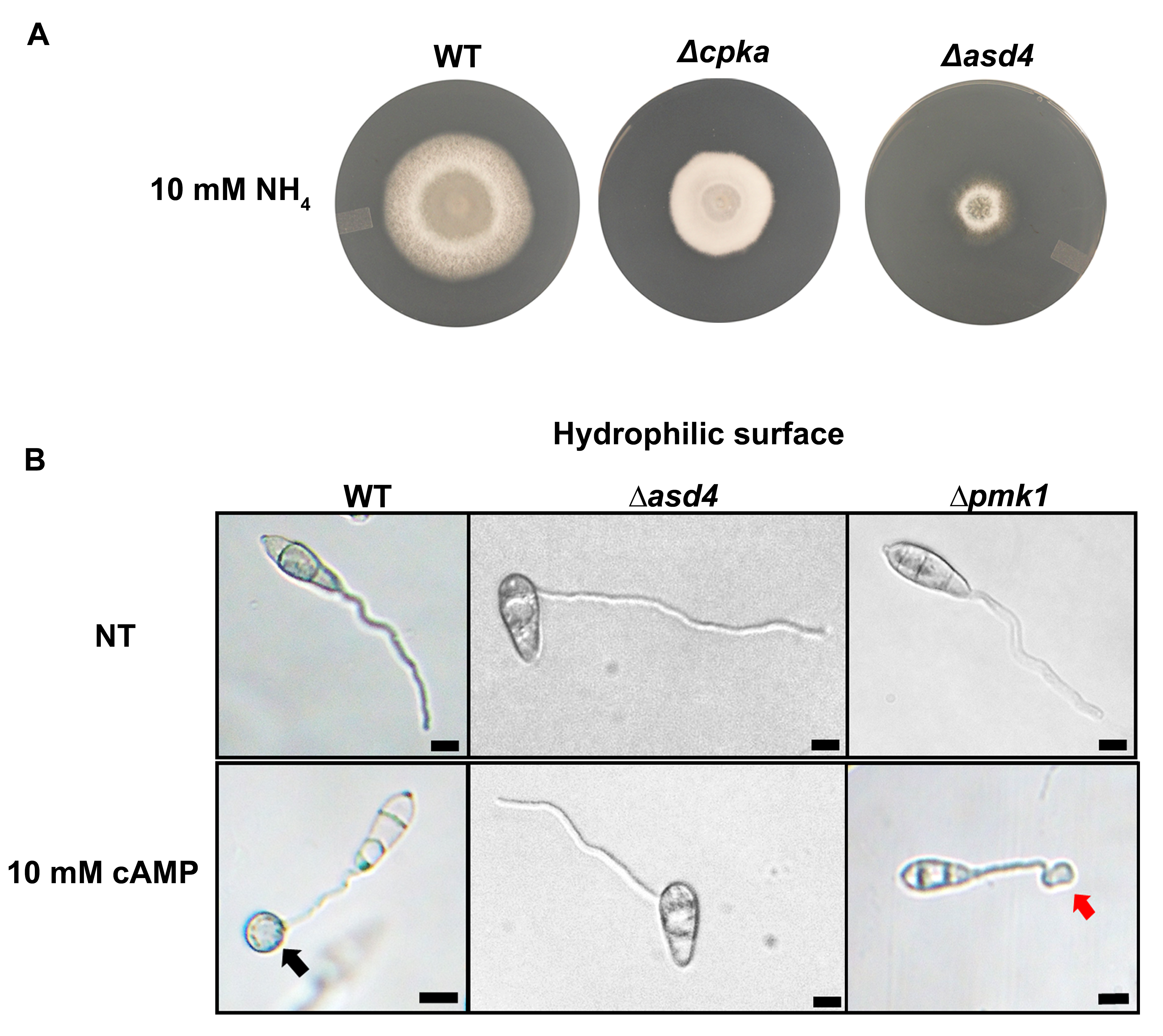

Supplement: S6 Fig — (A) Asd4 acts downstream but independently of cAMP/ PKA signaling. Unlike Δasd4, Δcpka mutant strains were not significantly reduced in growth on GMM with 10 mM NH4 +, indicating Asd4 is not under cAMP/ PKA signaling control. (B) Asd4 regulates appressorium formation upstream of Pmk1. Treatment with 10 mM cAMP resulted in appressorium formation by WT on hydrophilic surfaces. Δpmk1 strains responded to cAMP by differentiating germ tube tips on hydrophilic surfaces that hooked and swelled but did not form appressoria, indicating Pmk1 functions downstream of cAMP/PKA signaling [16]. Δasd4 mutant strains did not respond to cAMP treatment on hydrophilic surfaces and their germ tube tips did not differentiate, indicating Asd4 functions upstream of Pmk1. Appressoria are denoted by black arrow. Red arrow denotes differentiated germ tube tips that do not progress beyond hooking and swelling. Bar is 10 μm. Images were made at 24 hpi. NT = no treatment. (TIF) [file ppat.1004851.s006.tif]
