# Supplementary material for: GATA-Dependent Glutaminolysis Drives Appressorium Formation in Magnaporthe oryzae by Suppressing TOR Inhibition of cAMP/PKA Signaling
Source: PLoS Pathog. 2015 Apr 22;11(4):e1004851. doi: 10.1371/journal.ppat.1004851 (PMC4406744; doi:10.1371/journal.ppat.1004851)
Supplement: S1 Table — (DOCX) [file ppat.1004851.s007.docx]

**Table S1**. The colony size (in mm) of *Δasd4* mutant strains is significantly reduced (p < 0.05) compared to WT following ten days growth on defined minimal media with 1% (w/v) glucose as the sole carbon source and the indicated final concentrations of sole nitrogen sources.

| **Growth media** | **Strains** | | | |
| --- | --- | --- | --- | --- |
|  | **WT** | | ***Δasd4*** | |
|  | **Radial growth (mm)**^d^ | **SD**^e^ | **Radial growth (mm)**^d^ | **SD**^e^ |
| 10mM NH_4_^+^ ^a,^ * | 48.3 | 2.0 | 32.0 | 1.3 |
| 10mM Gln * | 44.0 | 1.5 | 21.0 | 2.0 |
| 1mM Gln * | 49.0 | 2.0 | 23.0 | 1.5 |
| 0.5mM Gln * | 47.0 | 3.0 | 24.0 | 2.0 |
| 10mM Glu * | 45.0 | 1.0 | 26.0 | 0.0 |
| 1mM Glu * | 48.3 | 0.0 | 22.6 | 2.2 |
| 0.5mM Glu * | 44.6 | 2.0 | 27.0 | 1.0 |
| 10mM Pro * | 52.0 | 1.0 | 32.0 | 0.0 |
| 1mM Pro * | 53.0 | 1.5 | 32.0 | 3.3 |
| 10mM Orn * | 46.0 | 2.0 | 34.0 | 1.0 |
| 1mM Orn * | 54.3 | 1.0 | 33.0 | 1.6 |
| 10mM Urea * | 45.0 | 2.0 | 28.2 | 2.6 |
| 1mM Urea * | 53.0 | 3.0 | 32.3 | 2.6 |
| 10mM Arg * | 55.3 | 2.0 | 30.0 | 0.0 |
| 10mM Asp * | 45.2 | 1.5 | 25.2 | 1.6 |
| 1mM Asp * | 57.3 | 2.0 | 33.0 | 1.0 |
| 0.5mM Asp * | 54.2 | 1.0 | 34.0 | 0.0 |
| 10mM Asn * | 47.5 | 1.5 | 21.6 | 1.2 |
| 1mM Asn * | 57.0 | 1.0 | 37.0 | 0.0 |
| 0.5mM Asn * | 56.6 | 2.0 | 33.6 | 1.3 |
| 10mM Ser * | 54.0 | 1.0 | 33.0 | 1.0 |
| 10mM NO_3_^- b,^* | 46.2 | 2.0 | 35.0 | 1.3 |
| 10mM GABA^c,^ * | 57.0 | 0.5 | 39.0 | 0.0 |
| 1mM GABA * | 56.0 | 2.2 | 37.6 | 1.2 |
| 0.5mM GABA * | 56.0 | 1.3 | 32.0 | 0.0 |
| 10mM Ala * | 57.0 | 1.0 | 39.3 | 1.6 |
| 10mM His * | 60.0 | 2.2 | 31 | 0.0 |

^a^NH_4_^+^ = ammonium

^b^NO_3_^-^ = nitrate

^c^GABA = gamma aminobutyric acid

^d^ Values correspond to the average of three independent repetitions.

^e^SD= Standard deviation

* p value < 0.05
