# Supplementary material for: GATA-Dependent Glutaminolysis Drives Appressorium Formation in Magnaporthe oryzae by Suppressing TOR Inhibition of cAMP/PKA Signaling
Source: PLoS Pathog. 2015 Apr 22;11(4):e1004851. doi: 10.1371/journal.ppat.1004851 (PMC4406744; doi:10.1371/journal.ppat.1004851)
Supplement: S2 Table — (DOCX) [file ppat.1004851.s008.docx]

| **Strains** | **Radial growth (mm) on minimal media**^c^ | | | | | |
| --- | --- | --- | --- | --- | --- | --- |
|  | 10mM Gln only^a^ * | 1mM Gln only * | 10mM Gln +10mM NH_4_^+^* | 10mM GABA^b^ +10mM NH_4_^+^* | 1% xylose +10mM Gln* | 1% xylose +10mM Glu* |
| WT | 48.0 ± 0.5 | 47.0 ± 1.3 | 40.0 ± 1.0 | 45.0 ± 1.0 | 42.0 ± 2.0 | 42.6 ± 2.2 |
| *Δasd4* | 23.0 ± 2.0 | 22.3 ± 2.0 | 18.6 ± 1.2 | 21.3 ± 1.0 | 23.0 ± 1.0 | 21.0 ± 1.0 |

**Table S2**. The colony size (in mm) of *Δasd4* mutant strains is significantly reduced (p < 0.05) compared to WT following ten days growth on defined minimal media with the indicated final concentrations of sole carbon and nitrogen sources.

^a^Gln only = glutamine as both a sole carbon and nitrogen source.

^b^GABA = gamma aminobutyric acid

^c^ Values correspond to the average of three independent repetitions.

* p value < 0.05

± is standard deviation
