# Supplementary material for: GATA-Dependent Glutaminolysis Drives Appressorium Formation in Magnaporthe oryzae by Suppressing TOR Inhibition of cAMP/PKA Signaling
Source: PLoS Pathog. 2015 Apr 22;11(4):e1004851. doi: 10.1371/journal.ppat.1004851 (PMC4406744; doi:10.1371/journal.ppat.1004851)
Supplement: S3 Table — (DOCX) [file ppat.1004851.s009.docx]

**Table S3**. Description of *Magnaporthe oryzae* genes analysed by quantitative RT-PCR in this study.

| **Locus** | ***M. oryzae* gene name** | **Reference** | **Molecular function** |
| --- | --- | --- | --- |
| MGG_06888 | *GLN1* | *This study* | Glutamine synthetase (GS) |
| MGG_14279 | *GLN2* | *This study* | ^a^ Glutamine synthetase (GS) |
| MGG_07187 | *GLT1* | *This study* | ^a^ Glutamate synthase (GOGAT) |
| MGG_08074 | *GDH1* | *This study* | ^a^ NADP-dependent glutamate dehydrogenase (NAD-GDH) |
| MGG_05247 | *MGD1* | 1 | ^a^ NAD-dependent glutamate dehydrogenase (NAD-GDH) |
| MGG_09222 | *RS2* | 2 | 40S ribosomal protein S2 |
| MGG_05673 | *RS3* | 2 | 40S ribosomal protein S3 |
| MGG_01062 | *ATG8* | 2 | Autophagy |

^a^ Putative function based on sequence homology.

1. Oh Y, Donofrio N, Pan H, Coughlan S, Brown DE, Meng S, et al. Transcriptome analysis reveals new insight into appressorium formation and function in the rice blast fungus *Magnaporthe oryzae*. Genome Biol. 2008; 9: R85.

2. Fernandez J, Marroquin-Guzman M, Wilson RA. Evidence for a transketolase-mediated metabolic checkpoint governing biotrophic growth in rice cells by the blast fungus *Magnaporthe oryzae*. PLOS Pathogens. 2014; 10 (9): e10004354. DOI: 10.1371/journal.ppat.1004354
