# Supplementary material for: GATA-Dependent Glutaminolysis Drives Appressorium Formation in Magnaporthe oryzae by Suppressing TOR Inhibition of cAMP/PKA Signaling
Source: PLoS Pathog. 2015 Apr 22;11(4):e1004851. doi: 10.1371/journal.ppat.1004851 (PMC4406744; doi:10.1371/journal.ppat.1004851)
Supplement: S4 Table — (DOC) [file ppat.1004851.s010.doc]

**Table S4**. Oligonucleotide primers used in this study.

| **Gene** | **Primer** | **Sequence 5’ – 3’** |
| --- | --- | --- |
| *hph*^1^ | Hyg-M13F^4^ | **CGCCAGGGGTTTTCCCAGTCACGAC** GTCGTGACTGGGAAAACCCTGGCG |
|  | Hyg-split | GGATGCCTCCGCTCGAAGTA |
|  | Hyg-M13R^4^ | **AGCGGATAACAATTTCACACAGGA** TCCTGTGTGAAATTGTTATCCGCT |
|  | Yg-split | CGTTGCAAGACCTGCCTGAA |
| *ILV1^2^* | M13F:IL^4^ | **CGCCAGGGGTTTTCCCAGTCACGAC**GTCGACGTGCCAACGCCACAG |
|  | ILSplit | AAGCATGTGCAGTGCCTTC |
|  | M13R:LV1^4^ | **AGCGGATAACAATTTCACACAGGA**GTCGACGTGAGAGCATGCTAA |
|  | LV1Split | CGCCCGGCCGACATCC |
| *Bar^3^* | M13F:BA^4^ | **CGCCAGGGGTTTTCCCAGTCACGAC**GTCGACAGAAGATGATATTGAAGGAG |
|  | BaSplit | GAGCCCAGTCCCGTCCG |
|  | M13R:AR^4^ | **AGCGGATAACAATTTCACACAGGA**CTAAATCTCGGTGACGGGCAGG |
|  | M13F:BA | CGCCCGGCCGACATCC |
| *GLN1* | GLN1-LF5’ ^5^ | ATGACAGGGTCTTGAAAGGGTCGT |
|  | GLN1-LF3’ ^5,4^ | **GTCGTGACTGGGAAAACCCTGGCG** TGACGGCGTTTAAGTGGGGAA |
|  | GLN1-RF5’ ^5,4^ | **TCCTGTGTGAAATTGTTATCCGCT**CGGTGAGCCCAACATCATCGT |
|  | GLN1-RF3’ ^5^ | CAGGTCTCCATGATAATGCCGGTA |
|  | GLN1-nesF ^5^ | TCCCTTTCCCCTAACATGGTATTTTC |
|  | GLN1-nesR ^5^ | TACCGGCATTATCATGGAGACCTG |
| *ASD4* | ASD4-LF5’ ^5^ | ATGCGACCCCATCCTCCAATATT |
|  | ASD4-LF3’ ^5, 4^ | **GTCGTGACTGGGAAAACCCTGGCG**CTGGGTAGCCTGGCCGTACAGAT |
|  | ASD4-RF5’ ^5, 4^ | **TCCTGTGTGAAATTGTTATCCGCT**CATGGCTTATTCGAATTTCTAATGATACC |
|  | ASD4-RF3’ ^5^ | CAAGATATTCAAGCAACCAACACCG |
|  | ASD4-nesF ^5^ | CCGTGCCAGGAGCTTGATTAGATC |
|  | ASD4-nesR ^5^ | GAGCGCCGCCAAGTCTATAGTTTTC |
| *MoFPR1* | MoFPR1-LF5’ ^5^ | TTAGTATCTGGTTGTGTGGCTTCATTG |
|  | MoFPR1-LF3’ ^5, 4^ | **GTCGTGACTGGGAAAACCCTGGCG**ACGTCGAGATGAATGGAAAAGGAA |
|  | MoFPR1-RF5’ ^5, 4^ | **TCCTGTGTGAAATTGTTATCCGCT** TCCTGCACGGCAATGGTAACTC |
|  | MoFPR1-RF3’ ^5^ | TCGGACCAGTCATGCTCTCTGTT |
|  | MoFPR1-nesF ^5^ | CACCTATCTCAACCTACCACCAACG |
|  | MoFPR1-nesR ^5^ | CTCGTATTGTCGCCAAAGTCGG |
| *GLN2* | GLN2-LF5’ ^5^ | GCGGTGAGGGCAAAACAG |
|  | GLN2-LF3’ ^5,4^ | **GTCGTGACTGGGAAAACCCTGGCG**CATTTCTGCTTGGGAGAGGTT |
|  | GLN2-RF5’ ^5,4^ | **TCCTGTGTGAAATTGTTATCCGCT**CAAGTTCAACCACCGCCAC |
|  | GLN2-RF3’ ^5^ | CGTTGGATGCCGGGC |
|  | GLN2-nesF ^5^ | CAGCCGTCTTTTTTGGATAGC |
|  | GLN2-nesR ^5^ | CCTTGGCGGCGCATTC |
| *TorA* | TorA-LF5’ ^5^ | GCATGACCGAGAGATTCCAGCAG |
|  | TorA-LF3’ ^5, 4^ | **GTCGTGACTGGGAAAACCCTGGCG**AGCGACAATCACCGGAGTGTTACTA |
|  | TorA-RF5’ ^5, 4^ | **TCCTGTGTGAAATTGTTATCCGCT**GAGACTTTGCCAAGATTCAAGGACC |
| **Gene** | **Primer** | **Sequence 5’ – 3’** |
| *TorA* | TorA-RF3’ ^5^ | CCGAGAGTGTTCAGGGCCAGC |
|  | TorA-nesF ^5^ | GGGCGGATTCCTTGACGAGC |
|  | TorA-nesR ^5^ | CCTTTGGAATAATGGGCACCG |
| *TUB1* | QRT-PCR b-tub F2 ^6^ | CGCGGCCTCAAGATGTCGT |
|  | QRT-PCR b-tub R2 ^6^ | GCCTCCTCCTCGTACTCCTCTTCC |
| *MoACT1* | MgActinF^6^ | AGCGTGGTATCCTCACTTTGCG |
|  | MgActinR^6^ | TCATCTTCTCTCGGTTGGACTTGG |
| *GLN1* | qGLN1F ^6^ | GGGCAGGCATAAGGAGCATATTG |
|  | qGLN1R ^6^ | GGTCTCCATGATAATGCCGGTAATC |
| *MGD1* | qMGD1 F ^6^ | GTTCTCAAGGACGCTCTGCCAAAC |
|  | qMGD1 R ^6^ | CTACTGAATCTGCGCCATCTTTGC |
| *GDH1* | qGDH1 F ^6^ | GTGGTGGTGTCGCCGTCTCTG |
|  | qGDH1 R ^6^ | TCACCCTGGGCCTGCATGG |
| *GLT1* | qGLT1F ^6^ | GTCAAAACGGCGCCGGG |
|  | qGLT1R ^6^ | CGACAGGTGCAACAGGCTTAACAG |
| *GLN2* | qGLN2F ^6^ | CAGGAGCACATTGCCGTCTACG |
|  | qGLN2R ^6^ | CGAAGCAGGTCTCCATGATGATACC |
| *RS2* | qRS2F ^6^ | GGTTGCCTCGCCCGCTG |
|  | qRS2R ^6^ | CGCTTGCCGTCCCTGAGG |
| *RS3* | qRS3F ^6^ | TCGTTCAGCCCGTCAGCCAA |
|  | qRS3R ^6^ | CTCCTGCTCCTCACCCTCACCC |
| *ATG8* | qATG8F ^6^ | CAGGTCGCCGAAGGTGTTCTC |
|  | qATG8R ^6^ | GCCACCATCGACAAGAAGAAGTACC |
| *ASD4* | ASD4-GF ^7,8^ | **TATAGGGCGAATTGGGTACTCAAATTGGTT**CGTGTACGTCTTCTTGCTCTTGTC |
|  | ASD4-GR ^7,8^ | **CCCGGTGAACAGCTCCTCGCCCTTGCTCAC**GACATCTTCGAGACGCCTTTTCAT |
|  | ASD4-nesGF ^8^ | CAGAATCGCCTGGAATACAGAGCT |
|  | ASD4-nesGR ^8^ | GACATCTTCGAGACGCCTTTTCAT |
| *GLN1* | qChipGLN1-F^9^ | CACAACCAGACCCTCAAGGAACAA |
|  | qChipGLN1-R^9^ | ACGATGATGTTGGGCTCACCG |

^1^Primers for *hygromycin B* resistance gene amplification [1]. ^2^ Primers for sulphonylurea resistance gene amplification [1].^3^ Primers for bialaphos resistance gene amplification [1]. ^4^M13F/M13R sequences for split marker gene deletion construct, highlighted in bold, are upstream of the gene specific sequences [1]. ^5^Primers for split marker deletion construct [1]. ^6^Primers used for qRT-PCR analysis. ^7^Nucleotide sequences of Native-C-FP and GFP-C-RP, highlighted in bold, added to the 5’-end of the promoter region and 3’-end of the open reading frame of the target gene, respectively [2]. ^8^Primers used for generation of the ASD4^GFP^ complementation strain [2]. ^9^Primers used to detect *GLN1* DNA following ChIP.

**Table S4**. Oligonucleotide primers used in this study *(Continued).*

1. Wilson RA, Gibson RP, Quispe CF, Littlechild JA, Talbot NJ. An NADPH-dependent genetic switch regulates plant infection by the rice blast fungus. Proc Natl Acad Sci USA. 2010; 107: 21902-7. doi: 10.1073/pnas.1006839107

2. Zhou X, Li G, Xu JR. Efficient approaches for generating GFP fusion and epitope-tagging constructs in filamentous fungi. In *Fungal Genomics*. Xu, J.R. and Bluhm, B.H. (eds). Humana Press. 2011; 722**:** 199-212.
